# Tackling unintended consequences of grazing livestock farming: Multi-scale assessment of co-benefits and trade-offs for water pollution mitigation scenarios

**DOI:** 10.1016/j.jclepro.2022.130449

**Published:** 2022-02-15

**Authors:** Yusheng Zhang, Bruce Griffith, Steve Granger, Hadewij Sint, Adrian L. Collins

**Affiliations:** Sustainable Agriculture Sciences, Rothamsted Research, North Wyke, Okehampton, Devon, EX20 2SB, UK

**Keywords:** Livestock agriculture, Water quality, Gaseous emissions, Soil quality, Best management

## Abstract

A farm-to-landscape scale modelling framework combining regulating services and life cycle assessment mid-point impacts for air and water was used to explore the co-benefits and trade-offs of alternative management futures for grazing livestock farms. Two intervention scenarios were compared: one using on-farm interventions typically recommended following visual farm audits (visually-based) and the other using mechanistical understanding of nutrient and sediment losses to water (mechanistically-based). At farm scale, reductions in business-as-usual emissions to water of total phosphorus (TP) and sediment, using both the visually-based and mechanistically-based scenarios, were <5%. These limited impacts highlighted the important role of land drains and the lack of relevant on-farm measures in current recommended advisory lists for the soil types in question. The predicted impacts of both scenarios on free draining soils were significantly higher; TP reductions of ∼9% (visually-based) and ∼20% (mechanistically-based) compared with corresponding respective estimates of >20% and >35% for sediment. Key co-benefits at farm scale included reductions in nitrous oxide emissions and improvements in physical soil quality, whereas an increase in ammonia emissions was the principal trade-off. At landscape scale, simulated reductions in business-as-usual losses were <3% for both pollutants for both scenarios. The visually-based and mechanistically-based scenarios narrowed the gaps between current and modern background sediment loads by 6% and 11%, respectively. The latter scenario also improved the reduction of GWP100 relative to business-as-usual by 4%, in comparison to 1% for the former. However, with the predicted increase of ammonia emissions, both eutrophication potential and acidification potential increased (e.g., by 7% and 14% for the mechanistically-based scenario). The discrepancy of on-farm intervention efficacy across spatial scales generated by non-agricultural water pollutant sources is a key challenge for addressing water quality problems at landscape scale.

## Highlights

• Monitoring data frames visually- and mechanistically-based intervention scenarios.
• Farm to landscape modelling explores mitigation scenario co-benefits/trade-offs.
• Farm scenarios reduced total phosphorus (TP) and sediment by <5% on drained soils.
• Farm scenarios reduced TP and sediment by up to ∼20% and >35% on free draining soils.
• Reductions in nitrous oxide emissions are the main co-benefit at farm and landscape scales.

## Graphical abstract

**Figure:**
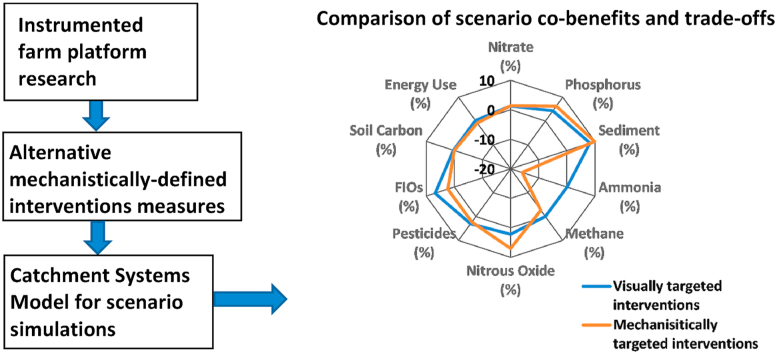

## 1 Introduction

Livestock farming sustains around 1.3 billion people worldwide and accounts for an estimated 14.5% of greenhouse gas emissions (HLPE, 2016). Overstocking and modern intensive management are known to damage soil structure, cause erosion and reduce biodiversity (e.g., Sansom, 1999). Modern livestock farming can therefore result in various unintended environmental consequences for the hydrosphere, pedosphere, atmosphere and biosphere. Diffuse water pollution from agriculture (DWPA) not only reduces nutrient use efficiency at farm scale, but also leads to environmental damage and economic costs at catchment scale.

The mitigation of DWPA is a complex challenge (Patterson et al., 2013). Efforts are testing new on-farm measures at experimental sites (Rovira et al., 2020), developing decision support tools for (Drohan et al., 2019), and surveying farmer attitudes to interventions (Inman et al., 2018). To generate timely and more holistic assessments at policy relevant scales, it is useful to integrate current evidence and expert opinion on the applicability and efficacy of on-farm interventions (Cuttle et al., 2016) and process understanding on key sources and pathways for diffuse pollution delivery from land to water (Forber et al., 2017) with modelling (e.g., Zhang et al., 2017). Importantly, modelling delivers several advantages including explicit consideration of co-benefits and trade-offs arising from different bundles of management interventions (Parkinson et al., 2019) and at multiple scales. Nevertheless, many existing models do not generate a fully comprehensive list of outcomes, lack some critical process representation, fail to compute outcomes across scales and are frequently designed to explore land cover change scenarios (Kanter et al., 2018).

Accordingly, we explored the potential outcomes of two on-farm mitigation scenarios for less favoured area (LFA) grazing livestock farms in SW England. One is based on current on-farm advice and the other uses mechanistic evidence on sediment and phosphorus (P) mobilization and delivery resulting from high resolution monitoring. A farm-to-landscape scale modelling framework was employed to assess the technically feasible co-benefits and trade-offs of each scenario relative to business-as-usual (BAU) at both farm and catchment scales. The hypotheses tested were: (i) the mechanistically-devised scenario will deliver greater reductions in water pollution than the visually-based scenario – addressing the evidence gap for assessment of mechanistically-defined management scenarios, rather than more conventional change in land cover scenarios; (ii) the efficacies of the intervention scenarios will be affected by soil drainage status dependent pathways – addressing the failure of many agri-system models to represent the assisted/preferential drainage pathway and its implications for intervention impacts explicitly; (iii) there are potential tradeoffs and co-benefits at farm scale and; (iv) non-agricultural water pollutant sources will generate a spatial mismatch of efficacy between farm and landscape scale – addressing the evidence gap for explicit representation of this mismatch in much existing modelling work.

## 2 Study site

**Fig. 1:**
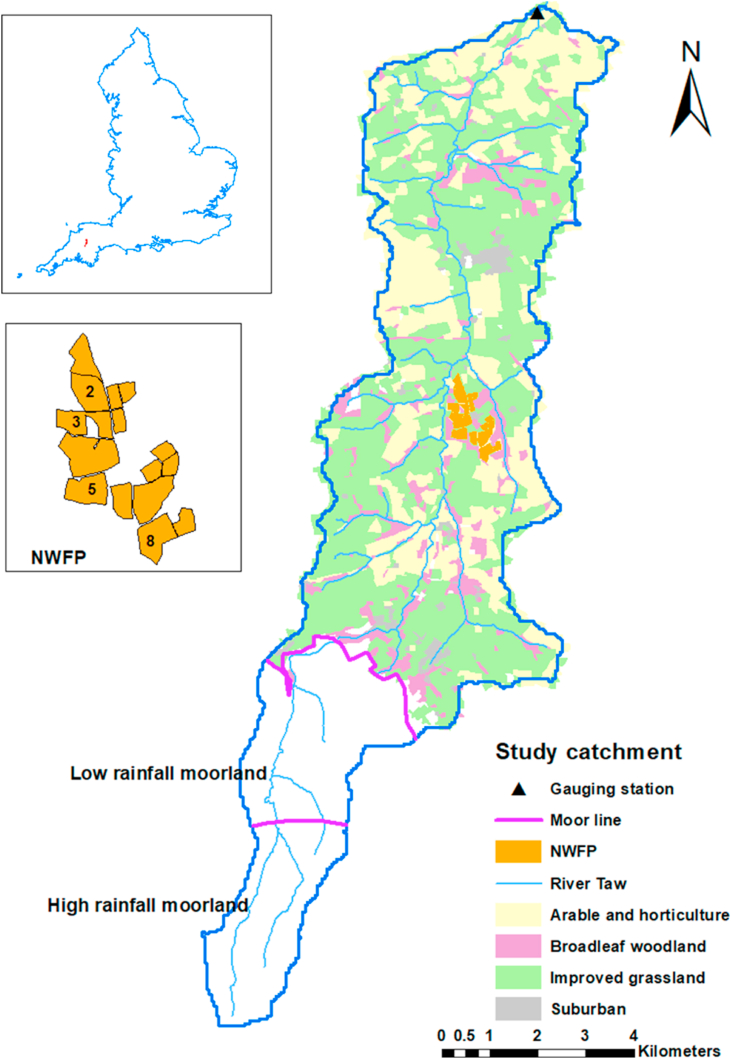
The upper River Taw catchment, showing its location in the UK, major land cover types, the North Wyke Farm Platform (NWFP) experimental farm and the outlet water quality monitoring station.

The study site (Fig. 1) encompasses multi-scale monitoring in the upper River Taw (∼71 km^2^). The North Wyke Farm Platform (NWFP) is in the upper reaches of the waterbody with hydrologically-isolated catchments ranging in size from 1.62 to 8.08 ha. Each catchment is equipped for monitoring rainfall and soil moisture, as well as discharge and water quality parameters (including turbidity and P fractions; orthophosphate - OP and TP) using flume laboratories (see details in Orr et al., 2016). The soils of the NWFP (Harrod and Hogan, 2008) predominantly belong to the Hallsworth (Dystric Gleysol) and Halstow (Gleyic Cambisol) series (Avery, 1980). Long-term (1980–2010) average annual rainfall for the site is 1044 mm.

The waterbody of the upper River Taw runs from the source of the river in the Dartmoor National Park to Taw Bridge, approximately 22 km downstream, where long-term monitoring is managed by the Environmental Agency. Climate is classified as oceanic (Köppen classification) and the land use can be divided into two: upland moorland comprising low intensity rough grazing grassland and a lowland agricultural area with a mixed grassland and cropping landscape. Long-term (1981–2010) average annual rainfall ranges between 1478.2 mm and 2468.1 mm for the upland area and between 957.5 and 1502.0 mm for the lowland agricultural area. While 15 soil series have been mapped in the study landscape, only six represent >5% of the catchment area on an individual basis (Table S1). The dominant soil types (69%) are free draining, but 21% of the land is heavy clay which needs to be artificially drained for grassland and arable use.

The upper River Taw has been assessed in conjunction with the chemical requirements of the EU Water Framework Directive (WFD) and judged to be failing due to elevated phosphate levels. Here, despite the landscape scale environmental objective being based on orthophosphate, it is important to acknowledge that water quality outcomes for phosphorus (P) will strongly reflect inputs of all forms of this nutrient, meaning that it is more appropriate to consider both OP and TP export and the capacity to improve management of all P forms (Lloyd et al., 2019). The upper River Taw has also been reported as failing to meet ‘good status’ due to the impacts of excessive sediment inputs on fish. Closure of the pollutant gaps for both P and sediment are therefore important environmental objectives for the study landscape.

## 3 Methodology

### 3.1 General approach

Our work integrated (Fig. S1) monitoring and modelling. Intensive field scale monitoring of water quality on an instrumented farm (NWFP) was used to extract mechanistic understanding to help frame modelling scenarios. A farm-to-landscape scale process-based modelling framework (CSM; catchment systems model) was used to compare and contrast the co-benefits and trade-offs of the modelled scenarios at both scales. Non-agricultural loads were used to correct the efficacy of interventions for reducing losses to water at landscape scale.

### 3.2 Processing of water quality data at field and landscape scales

Data on precipitation, flow and water quality, comprising turbidity, OP and TP, for four sub-catchments (catchments 2, 3, 5 and 8; Fig. S2) recorded at a 15-min resolution between late November 2016 and March 2019 (i.e., the main winter drainage period driving discharges of TP and sediment) were downloaded from the data portal for the NWFP (https://nwfp.rothamsted.ac.uk/). Further details are provided in Table S3.

For landscape scale, daily mean flow at the catchment outlet was downloaded from the UK National River Flow Archive (https://nrfa.ceh.ac.uk/data/station/info/50007). To avoid uncertainty from multiple rating curves in the period of record before 1998, the potential impacts of past water abstraction on low flow (up to 1999) and a known underestimation of flow (during 2000), only data between 2001 and 2017 were used without modification for further analysis and modelling. Monitored TSS and TP concentrations for the same periods were extracted from the Environmental Agency's Water Quality Data Archive (WIMS; Water Information Management System) for the same station (sampling ID SW-73030120; https://environment.data.gov.uk/water-quality/view/sampling-point/SW-73030120). For the period 2001–2017, there were 95 and 170 valid samples for TSS and TP, respectively. Monthly loads were estimated using the algorithms (Littlewood et al., 1998) detailed in supplementary information.

### 3.3 Application of the catchment systems model (CSM)

CSM is a modelling framework for assessing farm-to-landscape scale impacts of land use and management under BAU or alternative scenarios (Fig. S3). It has five interconnected modules. Module 1 is based on the intersection of rainfall, soil drainage status and farm system. The spatial variability of long-term (1981–2010) annual average rainfall (AAR) was mapped based on the HadUK-Grid data which provides a collection of gridded climate variables derived from the network of UK land surface observations at 1 km^2^ resolution (Meteorological Office, 2018). Soil drainage status was inferred from NatMap 1000 (National Soil Resources Institute, Cranfield University, UK) which summarizes soil series at 1 km^2^ resolution. Pedo-transfer functions (Zhang et al., 2017a, Zhang et al., 2017b) were used to assign Hydrology of Soil Types class and soil drainage status (free draining, drained for arable use or drained for both arable and grassland use) to the relevant NATMAP 1000 soil series. Input data on BAU farming activities were sourced from the June Agriculture Survey for 2016, licensed from the Department of Environment, Food and Rural Affairs (Defra). These data characterized farm types, crop areas and livestock counts, types and ages. The locations of the different farm types were provided by the Rural Payment Agency which, in turn, was used to generate the combinations of AAR, soil drainage status and robust farm types (RFTs; Defra, 2010). Farm and field management data were based on multiple year (2013–2017) averages of fertiliser application rates from the British Survey of Fertiliser Practice (BSFP, 2018). These rates were supplemented by some commercial farm business surveys in the study landscape. Based on the manure dressing data available in the BSFP, no significant change in manure spreading practices for spring crops, autumn crops or grassland was recorded between 2013 and 2017. The manure allocation scheme of Zhang et al., 2017a, Zhang et al., 2017b was thereby used. The farm scale module (Gooday and Anthony, 2010; Gooday et al., 2014; Zhang et al., 2017a, Zhang et al., 2017b) generated annual loadings of multiple pollutants to water and air and qualitative assessment of the relative impacts of BAU or the intervention scenarios on wider ecosystem services. To account for existing best management under BAU, prior implementation rates used in previous policy-focused work (Zhang et al., 2017a, Zhang et al., 2017b) were revised based on recent Defra farm practices surveys (FPS; https://www.gov.uk/government/collections/farm-practices-survey) and reported Countryside Stewardship (CS) uptake (https://naturalengland-defra.opendata.arcgis.com/datasets/countryside-stewardship-scheme-2016-management-options-england/data). Further background on the farm scale module is provided in supplementary information. More information on the grazing livestock farms in the study area is also included in supplementary information.

Module 2 scales out within the farming sector from farm to landscape scale using farm holding counts and types and their spatial distributions in the study catchment. Here, the farm type specific export coefficients from module 1 are used to generate the total emissions for BAU or the intervention scenarios using equation (1):

(1) $$C_{i}=\underset{k}{\sum }HC_{k}FL_{i\;k}$$

where *C*_*i*_ is the catchment scale total agricultural load for pollutant *i*, *k* represents a combination of AAR and soil drainage status, *HC* represent holding counts and *FL* represents the pollutant loads at farm scale. *C*_*i*_ was estimated using standard spatial join GIS procedures in ArcGIS 10.4.

Module 3 is used to estimate the spatial mis-match arising from nutrient and sediment loads from non-agricultural sources. These calculations utilize SEPARATE (Sector Pollutant AppoRtionment for the AquaTic Environment) reported in detail by Zhang et al. (2014) but using recently updated layers for the agriculture (2016–2017) and sewage treatment works (2013–2016) sources. This module includes estimates of nutrient and sediment losses to watercourses at WFD waterbody scale from direct atmospheric deposition, sewage treatment works, septic tanks, diffuse urban sources (urban and residential area runoff), storm tanks, combined sewer overflows and eroding channel banks.

Module 4 calculates temporal mis-match for the efficacy of on-farm intervention scenarios associated with landscape scale factors likely to generate a temporal lag in water quality outcomes (Fig. S3). This module was not applied in this study because the landscape was considered not to be heavily impacted by temporal lag factors. Module 5 is used for quantification of mid-point environmental impacts represented by eutrophication potential (EP), acidification potential (AP), global warming potential over 20 years (GWP20) or 100 years (GWP100), and sediment pressure potential (SP). EP, AP and GWP are founded on the characterization stage of Life Cycle Assessment (LCA); i.e. they are calculated by multiplying pollutant-specific characterization factors and the CSM annual export coefficients to aggregate the effects from different pollutants into common units (Heijungs et al., 1992) for the relevant impact categories. The characterization factors used (Table S2) are taken from IPCC 2013 (Myhre et al., 2013) for GWP20 and GWP100 and CML-IA 2016 (Oers, 2015) for EP and AP.

SP is based on the relative exceedance of the critical annual sediment load (*CL)* determined by catchment specific erosion risk, slope and land cover (Foster et al., 2011; Collins et al., 2021). Accordingly, using the total load for BAU (*BL*) and the intervention scenarios (*SL*), SP is calculated using equation (2):

(2) $$\frac{BL-SL}{SL-CL}*100$$

Taking the impacts for BAU as the baseline (*BP*), the relative changes resulting from the intervention scenarios (*IS*) were calculated using equation (3):

(3) $$\frac{BP-IS}{BP}*100$$

Predictions of pollutant emissions using the model routines from module 2 have been evaluated at field, catchment and national scale in the UK (Comber et al., 2013). For water, such evaluation has been reported by Collins et al. (2016) and Zhang et al. (2017b) using PARCOM (1991–2010) monitoring data, by Zhang et al. (2017a) using Harmonised Monitoring Scheme data (1980–2010) at 33 sites across England, and by Collins et al. (2021) using field scale monitored losses (2012–2016) on the NWFP. For air, methane and nitrous oxide emissions from agriculture predicted using the calculations embedded in CSM have been found to agree strongly with the UK GHG inventory dataset (Zhang et al., 2017b).

## 4 Results

### 4.1 Evaluation of CSM predictions for P and sediment losses to water

At field scale on the NWFP, the estimated specific loads from the four flumes used in this work ranged from 153 to 245 kg ha^−1^ for sediment and 0.45–0.65 kg ha^−1^ for TP. The corresponding estimates for grazing model farms on drained soils similar to the NWFP in CSM were 159–258 kg ha^−1^ for sediment and 0.40–0.50 kg ha^−1^ for TP. While it is advisable to be cautious in assessing model performance using the short temporal span of the monitored data, the comparison clearly suggests that CSM is providing robust predictions. At landscape scale, comparison of the CSM combined agricultural and non-agricultural sediment loads against WIMS data suggests a poorer fit, although the latter data will be heavily biased by the routine but infrequent sampling. Here, it is noteworthy that the estimated specific suspended sediment load of 31 t km^2^, using CSM, is in agreement with the mapped sediment yield of 35.8 t km^2^ (Cooper et al., 2008). The CSM predicted TP loss from all landscape sources (5.03 t) was in the range of the WIMS monitoring data (4.90–9.57 t) at the study landscape outlet monitoring site.

### 4.2 Mechanistic understanding on water pollutant transfers and measure selection for intervention scenarios

**Fig. 2:**
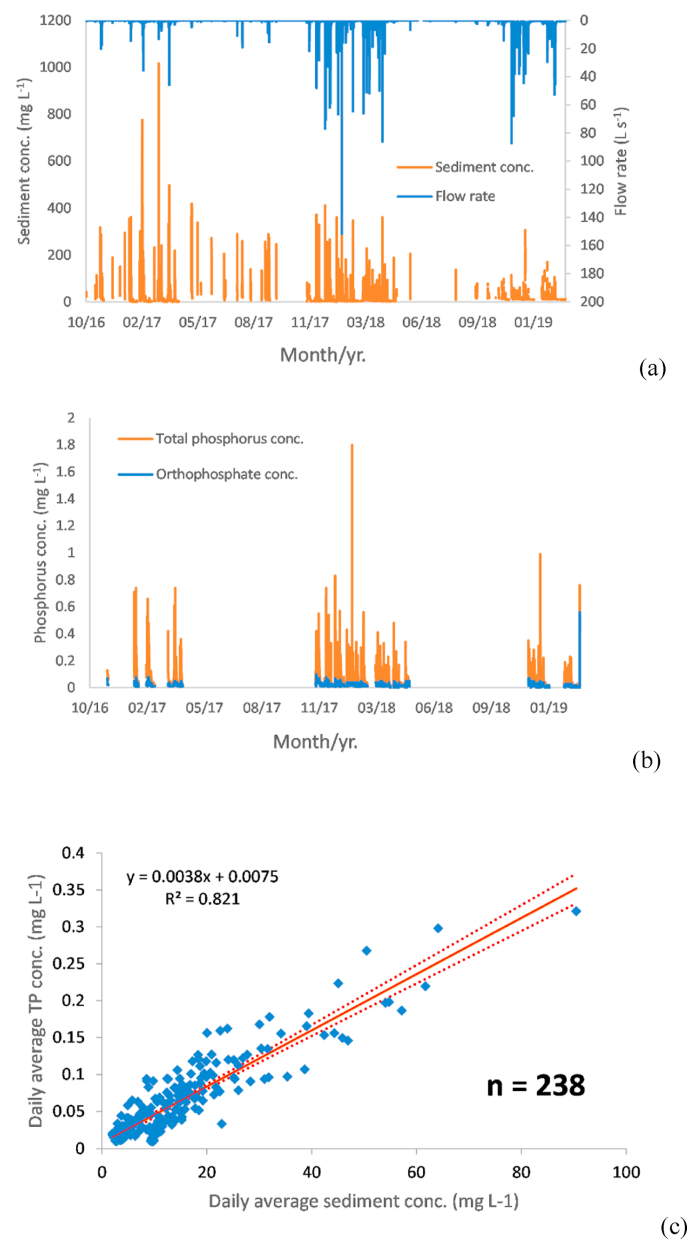
Monitored 15-min flow and sediment concentrations (a); 15-min orthophosphate (OP) and total phosphorus (TP) concentrations (b); and daily average concentrations of sediment and TP for NWFP field scale catchment 5 (c). In (c), the solid line is the fitted regression line and the dashed lines are the 95% confidence intervals.

Summary statistics of the flow rates and pollutant concentrations for the four NWFP field scale catchments used in this work are presented in Table S3. To illustrate their temporal trends throughout the monitoring period, 15-min resolution data from catchment 5 are shown in Fig. 2a and b. High flows generally correspond with high OP, TP and sediment concentrations, but the magnitude of change relative to baseflow conditions is much smaller in the case of OP (Fig. 2b). The observed ranges for all four NWFP catchments were 0.01 mg L^−1^ to 0.16 mg L^−1^ for OP, 0.01 mg L^−1^ to 1.80 mg L^−1^ for TP and 0.9 mg L^−1^ to nearly 800 mg L^−1^ for sediment. The marked differences in the magnitudes of the monitored OP and TP concentrations indicate the dominance of the particulate phosphorus (PP) fraction. Monitoring data from four field scale catchments on the NWFP clearly demonstrates that P loss to water from this ruminant livestock grazed farm platform is significantly related to winter storm-associated sediment transport. This relationship becomes even more pronounced when the daily averages are plotted (Fig. 2c). Plots for the other three NWFP field scale catchments also yielded strong positive relationships (see Figs. S4–S6). Here, the estimated slope coefficients range from 0.0032 to 0.0057, and the corresponding r^2^ values are mostly >0.7.

Currently, visual farm audits, commonly referred to as ‘walkover surveys’ (Reaney et al., 2019), are used to collect information on visually obvious problems for pollution loss to water which, in turn, is supplemented by follow-up visits to discuss and shortlist mitigation measures with farmers. This approach relies on limited farm visits and visual evidence, such as poaching damage. Pulley and Collins (2019), however, found no strong correlations between visually obvious poaching damage on the NWFP and sediment export measured at the flumes. Instead the strongest correlation was detected between sediment export and catchment area, showing that field-wide interventions, rather than highly spatially targeted interventions should be recommended. Since our monitored data demonstrate that P export from the NWFP is primarily controlled by sediment export, we have assumed that field-wide mitigation measures are also most relevant here. Accordingly, we modelled and compared the impacts of two mitigation scenarios. The first comprised measures typically recommended to address visually obvious poaching damage, whereas the second comprised field-wide measures more reflective of the mechanistic understanding of sediment and P export to water on the NWFP. The former scenario includes moving feeder rings at regular intervals, constructing troughs with a concrete base, re-siting gateways away from high risk areas, managing farm tracks and establishing riparian buffers. The latter scenario includes reducing the length of the grazing season, locating out-wintered stock away from watercourses, reducing field stocking rates when soils are wet, loosening compacted soil layers in grass fields and using correctly inflated low ground pressure tyres. Readers are referred to the section in the supplementary information document to understand the uptake rates, under BAU, of the on-farm measures included in the two modelled scenarios. Both scenarios assumed 100% uptake of the shortlisted farm management interventions but, did not include any structural changes to the grazing livestock farms (e.g., in stocking densities or land cover).

### 4.3 Modelled scenario efficacies at farm scale

Estimated specific loadings for the key farm types and different soil drainage classes in the study landscape are provided in Table 1. The simulated data shows that the specific loadings are affected by soil drainage status and farm type. In the case of nitrate loadings to water, the main driver is farm type. Intensive farming types, including cereal, dairy and mixed farms generally have higher specific loadings than those simulated for the relatively extensive farm types, such as LFA grazing or general cropping. For sediment and P, the main controlling factor is soil drainage status wherein even more significant differences are observed between free draining and drained soil classes (Table 1). It is widely recognized that subsurface drains are an efficient delivery pathway of sediment and associated pollutants (e.g. Foster et al., 2003). Specific loadings from drained soil for arable and grassland use are generally double the corresponding loadings from the free draining soils in the study landscape (Table 1). As expected, nitrous oxide emissions are highest on the intensively managed livestock farms, i.e. dairy farms, followed by the other livestock farms and then cropping farms (Table 1). Collectively speaking, variations in multiple specific farm scale pollutant loadings underscore the unintended consequences of modern farming practices and illustrate that these losses are not wholly driven by intrinsic environmental conditions, but instead, BAU farm management.

**Table 1:** Estimated BAU (average for 1981–2010) specific pollutant loadings to water and air at farm scale in the upper River Taw catchment.

Farm type	Soil drainage status	Nitrate	Total phosphorus - PO_4_–P	Sediment	Nitrous oxide^a^	FIOs^b^
		kg NO_3_–N ha^−1^	kg P ha^−1^	kg ha^−1^	kg N_2_O ha^−1^	10^9 cfu ha^−1^
Cereal	free draining	28.9	0.5	390	5.4	12
Cereal	drained for arable	32.4	1.7	1305	5.6	16
Cereal	drained for grassland	26.0	2.1	1366	5.3	29
General cropping	free draining	8.3	0.2	132	5.7	0
General cropping	drained for arable	8.8	0.4	226	5.7	0
General cropping	drained for grassland	6.4	1.2	603	5.6	0
LFA grazing	free draining	17.0	0.3	114	8.0	170
LFA grazing	drained for arable	18.0	0.4	159	8.0	226
LFA grazing	drained for grassland	15.2	1.5	512	7.9	380
Lowland	free draining	20.5	0.3	138	8.8	245
Lowland	drained for arable	21.8	0.5	258	8.9	325
Lowland	drained for grassland	18.5	1.8	617	8.7	548
Dairy	free draining	48.2	0.3	70	12.9	40
Dairy	drained for grassland	27.5	1.8	422	12.0	102
Mixed	free draining	27.5	0.5	306	8.0	84
Mixed	drained for arable	30.6	1.3	913	8.1	113
Mixed	drained for grassland	25.5	2.2	1123	7.9	194

a Emissions of methane and ammonia do not vary with soil drainage status in the CSM framework.

b Faecal indicator organisms.

**Fig. 3:**
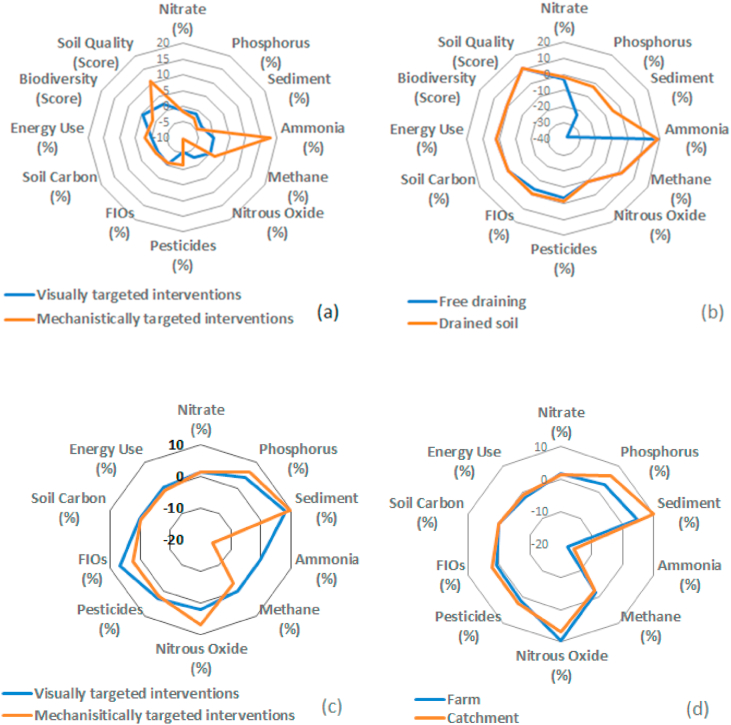
Simulated average efficacies (relative to business-as-usual - BAU) at multiple scales: (a) farm scale (less favoured area - LFA farms) comparison of the visually- and mechanistically-based intervention scenarios for more sustainable agriculture; (b) farm scale (LFA farms) comparison of the mechanistically-based scenario on free draining versus heavy drained soils in the study landscape; (c) landscape scale comparison of the visually-based and mechanistically-based scenarios in the upper River Taw catchment; (d) comparison of the mechanistically-based scenario at farm (LFA farms) and landscape scales.

The two intervention scenarios reflected visually-based or mechanistically-based lists of on-farm measures most relevant to the livestock farm types on drained soils in the study area, since these share similar environmental settings and management practices to the NWFP from which the mechanistic understanding is derived. Their efficacy on livestock farms on free draining soils in the study area was however, also evaluated for comparative purposes. The average farm scale efficacies across different rainfall conditions on drained soils for both intervention scenarios are illustrated in Fig. 3a. Positive values indicate either a percentage reduction or qualitative improvement in BAU annual loadings or scores. Negative values indicate pollution swapping or trade-offs. Fig. 3a shows that whilst the implementation of the mechanistically-based scenario performed marginally better than the visually-based alternative, both scenarios were estimated to be very limited with annual sediment and P load reductions of <5%. However, Fig. 3a also illustrates that there could be some noticeable co-benefits arising from the visually-based, but especially the mechanistically-based scenario, including reductions in BAU annual nitrous oxide emissions (3% and 10%, respectively) and improvements in soil physical quality (2% and 11%, respectively), but that these would be achieved at the expense of slightly increased energy use (2%) and with an 18% increase (for both intervention scenarios) in annual ammonia emissions. Fig. 3b illustrates the average efficacies of the two intervention scenarios for LFA farms on free draining and drained soils in the study area. Here, the predicted efficacy for reductions in agricultural sediment (>30%) and P (>20%) annual loadings relative to BAU, on free draining soils were substantially higher than those for the heavier drained soils. The remaining impacts on emissions to water or air and additional services were very similar to each other (Fig. 3b). Non-parametric comparisons of the predicted magnitude of impacts of the two intervention scenarios generally showed statistically significant results for free draining, but not for drained, soils (Table S6).

### 4.4 Scaling modelled scenario efficacies from farm to landscape scale

Projected reductions in BAU loadings associated with the implementation of the two intervention scenarios on all LFA farms within the study landscape are summarized in Table 2. These results suggest that the implementation of mechanistically-based on-farm scenario could result in more than double (2.21 times higher) the reduction of TP loads from LFA grazing farms relative to visually-based measures. The corresponding relative impact for reductions in BAU sediment loads is slightly less (1.83 times higher). Here, it is noteworthy that a focus on LFA grazing farms which are on heavy soils alone, could achieve very similar impacts as the relative annual load reductions only decrease from 2.21 to 2.18 for TP and from 1.83 to 1.66 for sediment. With the mechanistically-based future management scenario, the overall loadings of sediment and TP from LFA farms at landscape scale could be reduced by 13.7% and 7.1%, respectively.

**Table 2:** Estimated catchment-wide loadings of sediment and P from LFA grazing livestock farms in different environmental (rainfall/soil) settings and corresponding intervention impacts using the two scenarios.

Average annual rainfall(mm)	Soil drainage status	Landscape	Scenario efficacy (%)		Annual load reductions (kg)	
		wide loads (kg)	Visually-based	Mechanistically-based	Visually-based	Mechanistically-based
*Phosphorus*						
900–1200	Free draining	13.4	9.2	20.6	1.2	2.8
900–1200	Drained for grassland	638.7	1.4	2.9	8.6	18.7
1200–1500	Free draining	231.9	9.2	20.6	21.3	47.7
1200–1500	Drained for grassland	338.7	1.3	2.9	4.4	9.8
>1500	Free draining	61.8	9.3	20.5	5.7	12.7
Total					41.3	91.6
*Sediment*						
900–1200	Free draining	4405	22.2	37.6	976	1656
900–1200	Drained for grassland	204011	2.5	4.8	5161	9874
1200–1500	Free draining	89189	22.9	37.1	20406	33098
1200–1500	Drained for grassland	117389	2.8	4.8	3322	5635
>1500	Free draining	29204	24.1	37.2	7050	10875
Total					36916	61139

**Fig. 4:**
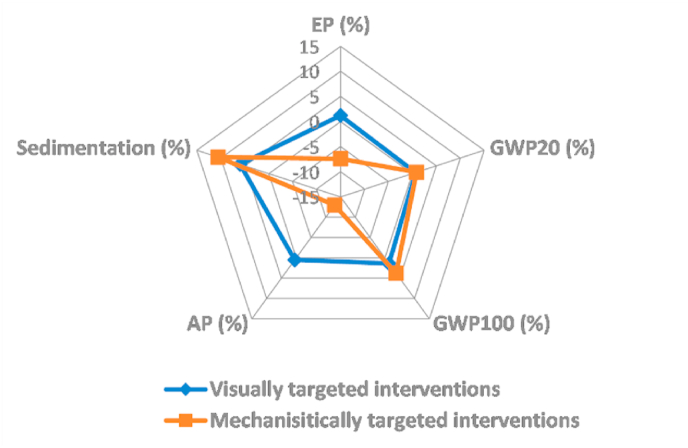
Simulated average efficacies (relative to BAU) of the visually- and mechanistically-based intervention scenarios for mid-point life cycle assessment (LCA) impact categories at catchment scale.

The wider implications of the scenarios at landscape scale are summarized in Fig. 3c. While the overall patterns remain comparable to those at farm scale, the positive effects forecast for sediment and TP loadings to water are enhanced slightly. This can be seen more clearly in Fig. 3d where the impacts of the mechanistically-based scenario at farm and landscape scales are compared. Herein, apart from the enhanced efficacies in reducing the agricultural loadings of sediment and TP delivered to watercourses, the unintended consequence of increased ammonia emissions at farm scale has become less of an issue at landscape scale. Estimated mid-point impacts of the intervention scenarios on LFA farms in the catchment are presented in Fig. 4. Both scenarios deliver greatest impacts on SP. The visually-based and mechanistically-based scenarios narrow the gaps between current and critical loads by 6% and 11%, respectively. The latter scenario will also improve the reduction of GWP100 relative to BAU by 4%, in comparison to 1% for the former. However, with the predicted increase of ammonia emissions, both EP and AP increase (e.g., by 7% and 14% for the mechanistically-based scenario). Readers are reminded that the inclusion of nitrate and ammonia in the calculation of EP projects the maximum potential impact assuming that both N and P are limiting factors. If only P loss is considered, EP will improve by 3% and 8% under the visually-based and mechanistically-based scenarios, respectively.

### 4.5 Scaling modelled efficacies for sediment and phosphorus loadings to water to assess spatial mismatch

Annual average loads from different catchment sources are presented in Table 3. The sum of the annual sediment loads from all listed sources exceeds the calculated loads based on the WIMS monitored flow and TSS concentration data at the study landscape outlet, but the estimated annual TP loads from all sources is more comparable to the WIMS monitored loads. The discrepancy for the former will in part, be driven by the infrequent monthly sampling undertaken by the Environment Agency at the catchment outlet. Regardless, the most important message from Table 3 is that landscape non-agricultural sources of sediment and P which will not benefit from increased future uptake of the measures in either modelled intervention scenario are likely to be significant contributors to the observed high concentrations at the landscape outlet monitoring site. This is especially the case for TP where sewage treatment plants alone are estimated to contribute 37% of the annual catchment scale load (Table 3). This result is also supported by the monitored TSS, OP and TP concentrations at the catchment outlet. Along with flow rates, their monthly average concentrations between 2000 and 2017 are shown in Table S5. The temporal patterns in Table S5 suggest that high P concentrations occur during summer low flow periods, i.e. June to September, wherein >90% of TP could be OP. In contrast, the OP fraction is only around 50% of the TP during the high flow winter period, and especially between December to February. The corrected landscape scale in-river impacts of the two intervention scenarios, assuming that the future management is only applied to LFA farms in the lowland area (Fig. 1), and taking account of the non-agricultural sources of TP are 1.2% (visually-based scenario) and 2.4% (mechanistically-based). For sediment, the corresponding respective estimates are 1.6% and 2.7%.

**Table 3:** Estimated annual sediment (TSS) and total phosphorus (TP) loads (tonnes) from cross sector sources in the upper Taw catchment.

Sources	TSS	TP
RLR farms^a^	1147	2.81
Low moor	117	0.16
High moor	101	0.11
Channel banks	786	0.02
Urban diffuse sources	7	0.02
Sewage treatment works	6	1.86
Storm tanks		0.09
Sceptic tanks		0.18
Direct deposition		0.05
Total	2164	5.03
WIMS^b^ derived	1533–1742	4.90–9.57

a Farms in the Rural Land Register (RLR) area of the study landscape covering all areas outside of the higher and lower rainfall upland moorland zones shown in Fig. 1.

b Water Information Management System.

## 5 Discussion

Optimizing sustainable food production continues to represent an important policy challenge. The UK decision to leave the EU and its Common Agricultural Policy (CAP), has provided both the opportunity and urgent need to revise agricultural policy. A significant shift here relates to the move away from area-based payments (£2.59 billion under CAP; HM Government, 2018) to a focus on delivering outcomes via public money for public goods. Outcomes include clean and plentiful water, clean air and a diverse and thriving plant and wildlife community. The goal for clean and plentiful water has rarely been observed in the outcomes from recent agri-policy schemes (Jones et al., 2017; Kay et al., 2012) which has resulted in both challenges to public purse expenditure (National Audit Office, 2010) and the need to redesign land management. In view of documented ongoing water quality failures, including in the study catchment, there remains a need to explore more nuanced future management scenarios since BAU uptake of best management practices is delivering limited benefits for reducing the excessive delivery of pollutants, including nutrients and sediment, from farmed land to water. Conventional high-resolution monitoring of water flow and multiple pollutant emissions on the NWFP (e.g., Pulley and Collins, 2019; Orr et al., 2016) provides valuable data from which to extract mechanistic understanding of the processes of pollutant delivery. This in turn, can be used to help identify and shortlist suitable on-farm measures to reduce pressures on watercourses arising from agricultural activities. Our work is novel on account of the combined assessment of multiple ecosystem services and LCA mid-point impacts resulting from two on-farm intervention scenarios, one of which, was defined using monitoring data from one of the most instrumented farm platforms in the world.

The upper River Taw landscape is experiencing water quality problems due to excessive P and sediment losses. Whereas riverine water quality compliance monitoring in the UK is focused on OP, consideration of TP fluxes is more appropriate since this more inclusive approach can be used to underpin the design of mitigation strategies targeting all key P sources (Lloyd et al., 2019). Monitoring evidence from the NWFP clearly underscores the episodic delivery of both pollutants in conjunction with rainfall-driven runoff and such delivery patterns have been noted by other research (Schilling et al., 2020; Sharpley and Withers, 1994). Whilst the ratio of OP to TP has been shown to vary substantially (Schilling et al., 2017), P export from poorly drained seasonally waterlogged soils is commonly dominated by TP (Jordan et al., 2012; Schilling et al., 2020). Again, monitoring data from the four field scale catchments on the NWFP is consistent with this wider understanding of P dynamics on poor draining soils. The dominance of TP to OP export from these soils underscores the significance of sediment delivery for P loss to water, especially since it can mobilize surface sediment sources enriched in P primarily as a result of fertilizer applications and direct excretion from grazing livestock (Withers et al., 2007). Importantly, PP losses could be expected to be elevated in conjunction with sediment losses which are in exceedance of modern background rates (Collins et al., 2021) In turn, recent mechanistic understanding on the principal forces mobilizing and delivering sediment on soils that are heavily poached on the seasonally waterlogged impermeable soils on the NWFP (Pulley and Collins, 2019) provided a basis for shortlisting measures for an intervention scenario. This was compared with BAU, alongside an alternative intervention scenario based on the types of measures typically recommended following visual audits on livestock farms.

For LFA farms, on poorly draining seasonally waterlogged soils similar to the NWFP, the mechanistically-defined scenario delivered marginally higher reductions in TP and sediment loss to water than the visually-based scenario. This supported hypothesis (i), but in both cases, the farm scale reductions amounted to <5% of BAU losses (Fig. 3a). Similar reductions are predicted for poorly drained soils across the different AAR bands at catchment scale. Here, the presence of artificial field drains on poorly draining seasonally waterlogged soils in the study area generates a preferential flow pathway which assists delivery of P and sediment alongside surface runoff (Heathwaite and Dils, 2000), including PP bound to colloids and fine clays (Foster et al., 2003). Very few policy-preferred measures (Cuttle et al., 2016) in the UK currently address the drain flow pathway, and those measures that are listed, including “allow grassland field drainage systems to deteriorate” are unpopular with farmers. Because of the reduced importance of the preferential flow pathway on free draining soils in the study area, the predicted impacts of the intervention scenarios on such soils across the AAR bands are significantly higher, amounting to TP reductions of ∼9% (visually-based scenario) and ∼20% (mechanistically-based) compared with corresponding respective estimates of >20% and >35% for sediment (Table 2). These results supported hypothesis (ii).

The most important co-benefits of the two intervention scenarios concern reductions in nitrous oxide emissions and improvements in physical soil quality, confirming hypothesis (iii). Positive impacts on the former are predicted at farm scale for LFA farms (Fig. 3a) and for LFA farms on both drained and free draining soils (Fig. 3b) because this scenario includes measures which improve stocking rates during wet periods and soil management to counter compaction problems. In 2017, nitrous oxide emissions from agriculture accounted for 8.6% of the UK total emissions (Brown et al., 2019) and have been earmarked for further reductions as the government pursues stricter environmental targets for greenhouse gases. Such co-benefits are not predicted at landscape scale (Fig. 3c) since the future management scenarios were only applied to LFA farms on both drained and free draining soils rather than all farms present in the upper Taw catchment. Readers are reminded that 2019 revisions to Intergovernmental Panel on Climate Change emission factors for agriculture were not used in this work. Similarly, the mechanistically-based scenario is predicted to deliver a greater co-benefit for soil quality on LFA farms than the visually-based scenario (Fig. 3b). This is because the former involves field-wide, rather than highly spatially-targeted (i.e. sub-field level), soil management to counter compaction and improve porosity and water-holding capacity. This co-benefit is well aligned to the aspirations in the 25 year environment plan to improve soil health by managing compaction (HM Government, 2018) which has been shown to be widespread (Newell-Price et al., 2013). The principal trade-offs of the mechanistic scenario at farm scale concerned an increase in energy use associated with soil compaction management and increased ammonia emissions, supporting hypothesis (iii).

Scaling up to the catchment scale in the study area underscores the importance of non-agricultural P sources. The combination of a high ratio of OP to TP and its timing at low flow periods is a good indicator of important contributions from steady and continuous discharge from non-storm related delivery landscape sources, e.g., known point sources including sewage treatment works and a creamery plant (Granger et al., 2017). Tappin et al. (2016) reached a similar conclusion after examining the phosphate trends in several sub-catchments of the River Taw and estimating the relative contributions from different sectors. The spatial mismatch generated by non-agricultural water pollutant sources is therefore a key challenge. This supported hypothesis (iv).

## 6 Limitations of the present study

All modelling has limitations. An important limitation in our work is that the interactions between interventions are assumed to be multiplicative in the absence of experimental evidence on the impacts of multiple combinations of best management practices on multiple outcomes. In some cases, the efficacy of individual interventions is based on expert judgment, since again, the empirical evidence base is lacking either for specific or all combinations of rainfall and soil type. Whilst the uptake rates of best management practices under BAU were based on a combination of strategic and bespoke surveys, ensuring this information is completely up to date for all farms in a locality is an ongoing challenge and uncertainty. The LCA work presented here calculated mid-point impacts for water based on pollutant delivery to watercourses and did not include any explicit consideration of pollutant fate during delivery through the river channel system. Nitrogen losses from agriculture include organic rather than just inorganic species, and there remains a need to incorporate such losses as relevant empirical evidence accumulates for different farm types in different geographies.

## 7 Practical implications of the present study

The main practical implication of this study concerns the urgent need to scope and support the implementation of engineering solutions for addressing the shortcomings of current best management options for reducing pollutant emissions to water on soils with artificial underdrainage. Here, options include cutting back field drains from watercourses to provide space for artificial wetlands which act as sumps and which can include reactive barrier materials such as woodchip (Lenhart et al., 2016). To help maximize the benefits of such point-based engineering solutions, options for ‘treatment-trains’ (Zhang et al., 2017a) wherein these end-of-pipe solutions are implemented in tandem with upslope interventions, should be scoped and encouraged. Importantly, however, the risks of trade-offs must be assessed and communicated to both farm advisors and farmers.

## 8 Conclusions and prospects

BAU best management in agriculture is not delivering the intended environmental outcomes meaning there is a need to explore alternative pathways to sustainable agriculture taking explicit account of co-benefits and trade-offs. This can be tackled with modelling. Our findings confirmed that a mechanistically-based scenario can deliver slightly greater reductions in pollutant emissions to water from LFA grazing livestock farms than a visually-based scenario, but that the impacts of both scenarios on pollutant emissions to water from such farm systems will be greater on free draining rather than seasonally-waterlogged soils with artificial drainage. The mechanistically-based scenario was forecast to deliver strong co-benefits for reductions in nitrous oxide emissions and improvements in soil quality, but with trade-offs for energy use due to compaction management and ammonia emissions. The spatial mismatch imparted by non-agricultural sources of water pollution reduces the efficacy of on-farm measures for water quality protection at landscape scale. One implication is that structural land cover changes will be required on LFA grazing livestock farms, and indeed other farm system types, to meet policy objectives. Future research will need to continue exploring mechanistically-based intervention scenarios but with an end point inclusive of emerging agricultural policy priorities including net zero.

## CRediT authorship contribution statement

**Yusheng Zhang:** Methodology, Validation, Writing – original draft, Writing – review & editing. **Bruce Griffith:** Investigation, Data curation. **Steve Granger:** Investigation. **Hadewij Sint:** Data curation. **Adrian L. Collins:** Funding acquisition, Project administration, Supervision, Methodology, Writing – original draft, Writing – review & editing.

## Declaration of competing interest

The authors declare that they have no known competing financial interests or personal relationships that could have appeared to influence the work reported in this paper.

## References

[bib1] Avery B.W. (1980).

[bib2] Brown P., Broomfield M., Cardenas L., Choudrie S., Jones L., Karagianni E., Passant N., Thistlethwaite G., Thomson A., Turtle L., Wakeling D. (2019). Ricardo Energy and Environment. The Gemini Building, Fermi Avenue Harwell Didcot Oxfordshire, OX11 0QR.

[bib3] BSFP (2018). British survey of fertiliser practice – fertiliser use on farm for the 2017 crop year. https://www.gov.uk/government/collections/fertiliser-usage.

[bib4] Collins A.L., Zhang Y.S., Winter M., Inman A., Jones J.I., Johnes P.J., Cleasby W., Vrain E., Lovett A., Noble L. (2016). Tackling agricultural diffuse pollution: what might uptake of farmer-preferred measures deliver for emissions to water and air. Sci. Total Environ..

[bib5] Collins A.L., Zhang Y., Upadhayay H., Pulley S., Granger S.J., Harris P., Sint H.M., Griffith B.A. (2021). Current advisory interventions for grazing ruminant farming cannot close exceedance of modern background sediment loss – assessment using an instrumented farm platform and modelled scaling out. Environ. Sci. Pol..

[bib6] Comber S.D.W., Smith S., Daldorph P., Gardner M.J., Constantino C., Ellor B. (2013). Development of a chemical source apportionment decision support framework for catchment management. Environ. Sci. Technol..

[bib7] Cooper D.M., Naden P., Old G., Laize C. (2008). Natural England Research Report NERR008.

[bib8] Cuttle S.P., Newell Price P., Harris D., Chadwick D.R., Shepherd M.A., Anthony S.G., Macleod C.J.A., Haygarth P.M., Chambers B.J. (2016). A method-centric ‘user manual’ for the mitigation of diffuse water pollution from agriculture. Soil Use Manag..

[bib9] Defra (2010).

[bib10] Drohan P.J., Bechmann M., Buda A., Djodjic F., Doody D., Duncan J.M., Iho A., Jordan P., Kleinman P.J., McDowell R., Mellander P.E., Thomas I.A., Withers P.J.A. (2019). A global perspective on phosphorus management decision support in agriculture: lessons learned and future directions. J. Environ. Qual..

[bib11] Forber K.J., Ockenden M.C., Wearing C., Holloway M.J., Falloon P.D., Kahana R., Villamizar M.L., Zhou J.G., Withers P.J.A., Beven K.J., Collins A.L., Evans R., Hiscock K.M., Macleod C.J.A., Haygarth P.M. (2017). Determining the effect of drying time on phosphorus solubilisation from three agricultural soils under climate change scenarios. J. Environ. Qual..

[bib12] Foster I.D.L., Chapman A.S., Hodgkinson R.M., Jones A.R., Lees J.A., Turner S.E., Scott M. (2003). Changing suspended sediment and particulate phosphorus loads and pathways in underdrained lowland agricultural catchments; Herefordshire and Worcestershire. U.K. Hydrobiologia.

[bib13] Foster I.D.L., Collins A.L., Naden P.S., Sear D.A., Jones J.I., Zhang Y. (2011). The potential for paleolimnology to determine historic sediment delivery to rivers. J. Paleolimnol..

[bib14] Gooday R., Anthony S. (2010).

[bib15] Gooday R., Anthony S., Chadwick D., Newell-Price P., Harris d., Duethmann D., Fish R., Collins A.L., Winter M. (2014). Modelling the cost-effectiveness of mitigation methods for multiple pollutants at farm scale. Sci. Total Environ..

[bib16] Granger S.J., Heaton T.H.E., Pfahler V., Blackwell M.S.A., Yuan H., Collins A.L. (2017). The oxygen composition of phosphate in river water and its potential sources in the Upper River Taw catchment, UK. Sci. Total Environ..

[bib17] Harrod T.R., Hogan D.V. (2008). The soils of North Wyke and Rowden. http://www.rothamsted.ac.uk/sites/default/files/SoilsNWRowden.pdf.

[bib18] Heathwaite A.L., Dils R.M. (2000). Characterising phosphorus loss in surface and subsurface hydrological pathways. Sci. Total Environ..

[bib19] Heijungs R., Guinée J.B., Huppes G., Lankreijer R.M., Udo de Haes H.A., Wegener Sleeswijk A. (1992). Environmental Life Cycle Assessment of Products: Guide and Backgrounds (Part 1) Leiden.

[bib20] HLPE (2016).

[bib21] HM Government (2018).

[bib22] Inman A., Winter M., Wheeler R., Vrain E., Lovett A., Collins A., Jones I., Johnes P., Cleasby W. (2018). An exploration of individual, social and material factors influencing water pollution mitigation behaviours within the farming community. Land Use Pol..

[bib23] Jones J.I., Murphy J.F., Anthony S.G., Arnold A., Blackburn J.H., Duerdoth C.P., Hawczak A., Hughes G.O., Pretty J.L., Scarlett P.M. (2017). Do agri-environment schemes result in improved water quality?. J. Appl. Ecol..

[bib24] Jordan P., Melland A.R., Mellander P.-E., Shortle G., Wall D. (2012). The seasonality of phosphorus transfers from land to water: implications for trophic impacts and policy evaluation. Sci. Total Environ..

[bib25] Kanter D.R., Musumba M., Wood S.L.R., Palm C., Antle J., Balvanera P., Dale V.H., Havlik P., Kline K.L., Scholes R.J., Thorton P., Tittonell P., Andelman S. (2018). Evaluating agricultural trade-offs in the age of sustainable development. Agric. Syst..

[bib26] Kay P., Grayson R., Philips M., Stanley K., Dodsworth A., Hanson A., Walker A., Foulgar M., McDonnell I., Taylor S. (2012). The effectiveness of agricultural stewardship for improving water quality at the catchment scale: experiences from an NVZ and ECSFDI watershed. J. Hydrol..

[bib27] Lenhart C., Gordon B., Gamble J., Current D., Ross N., Herring L., Nieber J., Petersen H. (2016). Design and hydrologic performance of a tile drainage treatment wetland in Minnesota. USA. Water.

[bib28] Littlewood I.G., Watts C.D., Custance J.M. (1998). Systematic application of United Kingdom river flow and quality databases for estimating annual river mass loads (1975–1994). Sci. Total Environ..

[bib29] Lloyd C.E.M., Johnes P.J., Freer J.E., Carswell A.M., Jones J.I., Stirling M.W., Hodgkinson R.A., Richmond C., Collins A.L. (2019). Determining the sources of nutrient flux to water in headwater catchments: examining the speciation balance to inform the targeting of mitigation measures. Sci. Total Environ..

[bib30] Meteorological Office (2018). HadUK-Grid Gridded Climate Observations on a 1km Grid over the UK for 1862-2017.

[bib31] Myhre G., Shindell D., Bréon F.-M., Collins W. (2013). Climate Change 2013: the Physical Science Basis. Contribution of Working Group I to the Fifth Assessment Report of the Intergovernmental Panel on Climate Change.

[bib32] National Audit Office (2010).

[bib33] Newell-Price J.P., Whittingham M.J., Chambers B.J., Peel S. (2013). Visual soil evaluation in relation to measured soil physical properties in a survey of grassland soil compaction in England and Wales. Soil Tillage Res..

[bib34] Oers L. van (2015). CML-IA database, characterisation and normalisation factors for midpoint impact category indicators. http://www.cml.leiden.edu/software/data-cmlia.html.

[bib35] Orr R.J., Murray P.J., Eyles C.J., Blackwell M.S.A., Cardenas L.M., Collins A.L., Dungait J.A.J., Goulding K.W.T., Griffith B.A., Gurr S.J., Harris P., Hawkins J.M.B., Misselbrook T.H., Rawlings C., Shepherd A., Sint H., Takahashi T., Tozer K.N., Whitmore A.P., Wu L., Lee M.R.F. (2016). The North Wyke Farm Platform: effect of temperate grassland farming systems on soil moisture contents, runoff and associated water quality dynamics. Eur. J. Soil Sci..

[bib36] Parkinson S., Krey V., Huppmann D., Kahil T., McCollum D., Fricko O., Byers E., Gidden M.J., Mayor B., Khan Z., Raptis C., Rao N.D., Johnson N., Wada Y., Djilali N., Riahi K. (2019). Balancing clean water-climate change mitigation trade-offs. Environ. Res. Lett..

[bib37] Patterson J.J., Smith C., Bellamy J. (2013). Understanding enabling capacities for managing the ‘wicked problem’ of nonpoint source water pollution in catchments: a conceptual. J. Environ. Manag..

[bib38] Pulley S., Collins A.L. (2019). Field-based determination of controls on runoff and fine sediment generation from lowland grazing livestock fields. J. Environ. Manag..

[bib39] Reaney S.M., Mackay E.B., Haygarth P.M., Fisher M., Molineux A., Potts M., Benskin C. McW.H. (2019). Identifying critical source areas using multiple methods for effective diffuse pollution mitigation. J. Environ. Manag..

[bib40] Rovira P., Ayala W., Terra J., García-Préchac F., Harris P., Lee M.R.F., Rivero M.J. (2020). The ‘Palo a Pique’ long-term research platform: first 25 Years of a crop–livestock experiment in Uruguay. Agronomy.

[bib41] Sansom A.L. (1999). Upland vegetation management: the impacts of overstocking. Water Sci. Technol..

[bib42] Schilling K.E., Kim S.-W., Jones C.S., Wolter C.F. (2017). Orthophosphorus contributions to total phosphorus concentrations and loads in Iowa Agricultural Watersheds. J. Environ. Qual..

[bib43] Schilling K.E., Streeter M.T., Seeman A., Jones C.S., Wolter C.F. (2020). Total phosphorus export from Iowa agricultural watersheds: quantifying the scope and scale of a regional condition. J. Hydrol..

[bib44] Sharpley A.N., Withers P.J. (1994). The environmentally-sound management of agricultural phosphorus. Fert. Res..

[bib45] Tappin A.D., Comber S., Paul J., Worsfold P.J. (2016). Orthophosphate-P in the nutrient impacted River Taw and its catchment (SW England) between 1990 and 2013. Environ. Sci.: Processes & Impacts.

[bib46] Withers P.J.A., Hodgkinson R.H., Adamson H., Green G. (2007). The impact of pasture improvement on phosphorus concentrations in soils and streams in an upland catchment in Northern England. Agric. Ecosyst. Environ..

[bib47] Zhang Y., Collins A.L., Murdoch N., Lee D., Naden P.S. (2014). Cross sector contributions to river pollution in England and Wales: updating waterbody scale information to support policy delivery for the Water Framework Directive. Environ. Sci. Pol..

[bib48] Zhang Y., Collins A.L., Jones J.I., Johnes P.J., Inman A., Freer J.E. (2017). The potential benefits of on-farm mitigation scenarios for reducing multiple pollutant loadings in prioritised agri-environment areas across England. Environ. Sci. Pol..

[bib49] Zhang Y., Collins A.L., Johnes P.J., Jones J.I. (2017). Projected impacts of increased uptake of source control mitigation measures on agricultural diffuse pollution emission to water and air. Land Use Pol..

