# Supplementary material for: Tackling unintended consequences of grazing livestock farming: Multi-scale assessment of co-benefits and trade-offs for water pollution mitigation scenarios
Source: J Clean Prod. 2022 Feb 15;336:130449. doi: 10.1016/j.jclepro.2022.130449 (PMC8837634; doi:10.1016/j.jclepro.2022.130449)
Supplement: Multimedia component 1 [file mmc1.docx]

**Tackling unintended consequences of grazing livestock farming: multi-scale assessment of co-benefits and trade-offs for water pollution mitigation scenarios**

Yusheng Zhang^1^*, Bruce Griffith^1^, Steve Granger^1^, Hadewij Sint^1^, and Adrian L. Collins^1^

^1^*Sustainable Agriculture Sciences, Rothamsted Research, North Wyke, Okehampton, Devon EX20 2SB, UK.*

^*^ Corresponding author: Yusheng Zhang

Email: yusheng.zhang@rothamsted.ac.uk

**Supplementary information**

**Table S0 List of abbreviations used and their descriptions**

| Abbreviation | Description |
| --- | --- |
| AAR | Average Annual Rainfall |
| AP | Acidification Potential |
| BAU | Business-As-Usual |
| CAP | Common Agricultural Policy |
| CSM | Catchment Systems Model |
| DWPA | Diffuse Water Pollution from Agriculture |
| EP | Eutrophication Potential |
| FIOs | Faecal Indicator Organisms |
| GHG | Greenhouse Gases |
| HM | Her Majesty |
| IPCC | Intergovernmental Panel on Climate Change |
| LCA | Life Cycle Assessment |
| LFA | Less Favoured Area |
| N | Nitrogen |
| NatMAP | National Soil Map |
| NWFP | North Wyke Farm Platform |
| OP | Orthophosphate Phosphorus |
| P | Phosphorus |
| PARCOM | Paris Commission |
| SP | Sediment pressure Potential |
| SW | Southweest |
| TP | Total Phosphorus |
| TSS | Total Suspended Solids |
| UK | United Kingdom |
| WFD | Water Framework Directive |
| WIMS | Water Information Management System |

Table S1: Distribution of soil series in the upper River Taw study catchment (data extracted from the NATMAP 1000 data product; http://www.landis.org.uk/data/nm1000.cfm).

| Soil series | Geology | Drainage status | Proportion (%) |
| --- | --- | --- | --- |
| Hallsworth | Drift from Palaeozoic shale or carboniferous and shale | Drained for arable and grassland | 21.6 |
| Neath | Carboniferous sandstone and shale | Free draining | 16.7 |
| Denbigh 1 | Palaeozoic slaty mudstone and siltstone | Free draining | 13.4 |
| Moor Gate | Granite and other acid igneous rock | Free draining | 10.1 |
| Halstow | Carboniferous shale | Drained for arable | 9.6 |
| Crediton | Permian and Carboniferous reddish breccia | Free draining | 8.1 |
| Crowdy | Blanket and basin peat | Free draining | 4.0 |
| Princetown | Granite and other acid igneous rock | Free draining | 3.7 |
| Laployd | Granite and other acid igneous rock | Free draining | 3.3 |
| Parc | Palaeozoic slaty mudstone and siltstone | Free draining | 2.7 |
| Alun | River alluvium | Free draining | 2.4 |
| Manod | Palaeozoic slate, mudstone and siltstone | Free draining | 2.4 |
| Teme | River alluvium | Free draining | 1.4 |
| Trushum | Basic igneous and metamorphic rock | Free draining | 0.3 |
| Winterhill | Blanket peat | Free draining | 0.2 |

Table S2: Characterisation factors used in the assessment of mid-point environmental impacts (CSM module 5).

|  | EP | GWP20 | GWP100 | AP |
| --- | --- | --- | --- | --- |
| Pollutant | kg PO_4_ eq. kg^-1^ | kg CO_2_ eq. kg^-1^ | kg CO_2_ eq. kg^-1^ | kg SO_2_ eq. kg^-1^ |
| Nitrate | 0.1 |  |  |  |
| Total phosphorus | 3.06 |  |  |  |
| Ammonia | 0.35 |  |  | 1.6 |
| Nitrous oxide |  | 264 | 265 | 0.5 |
| Methane |  | 84 | 28 |  |

Table S3: Summary statistics for monitored flow and pollutant concentrations for the NWFP field scale catchments (catchment numbers link to those in the inset image of the NWFP in Figure 1 in the main paper).

| Catchment | Summary | | Flow | Orthophosphate – PO_4_-P | Total Phosphorus - PO_4_-P | Sediment |
| --- | --- | --- | --- | --- | --- | --- |
| ID (area; ha) | statistics | (L S^-1^) | | (mg L^-1^) | (mg L^-1^) | (mg L^-1^) |
| 2 (6.65) | min | <MDL | | <MDL | <MDL | 5.0 |
|  | max | 98.32 | | 0.43 | 1.18 | 435.8 |
|  | median | 0.06 | | 0.02 | 0.05 | 10.0 |
|  | Q1 | 0.01 | | 0.01 | 0.03 | 6.9 |
|  | Q3 | 0.30 | | 0.03 | 0.09 | 15.7 |
|  | Samples | 75874 | | 16905 | 17149 | 29410 |
| 3 (6.62) | min | <MDL | | <MDL | <MDL | 3.8 |
|  | max | 89.67 | | 0.32 | 1.45 | 548.1 |
|  | median | 0.12 | | 0.02 | 0.03 | 8.4 |
|  | Q1 | 0.02 | | 0.01 | 0.02 | 6.1 |
|  | Q3 | 0.43 | | 0.02 | 0.07 | 14.0 |
|  | Samples | 80091 | | 19650 | 19650 | 38224 |
| 5 (6.54) | min | <MDL | | <MDL | <MDL | 0.1 |
|  | max | 151.10 | | 0.56 | 1.80 | 1016.6 |
|  | median | 0.09 | | 0.02 | 0.04 | 9.7 |
|  | Q1 | <MDL | | 0.01 | 0.02 | 4.2 |
|  | Q3 | 0.43 | | 0.03 | 0.08 | 16.2 |
|  | Samples | 75128 | | 24253 | 24408 | 29400 |
| 8 (7.02) | min | <MDL | | <MDL | <MDL | 0.1 |
|  | max | 125.66 | | 0.27 | 1.18 | 744.2 |
|  | median | 0.05 | | 0.02 | 0.03 | 8.9 |
|  | Q1 | <MDL | | 0.01 | 0.02 | 3.7 |
|  | Q3 | 0.25 | | 0.02 | 0.07 | 20.2 |
|  | Samples | 75994 | | 21600 | 21734 | 28254 |

<MDL means the concentration was below method detection limits

Table S4: Mapped spatial distribution of farm system types in the upper River Taw study catchment.

| Farm type | Average annual rainfall band (mm) | Soil drainage | Proportion |
| --- | --- | --- | --- |
| Cereal | 900 -1200 | Free draining | 0.62 |
| Cereal | 900 -1200 | Drained for arable and grassland | 0.38 |
| General cropping | 900 -1200 | Free draining | 0.29 |
| General cropping | 900 -1200 | Drained for arable and grassland | 0.35 |
| General cropping | 1200 - 1500 | Free draining | 0.29 |
| General cropping | > 1500 | Free draining | 0.06 |
| Dairy | 900 -1200 | Free draining | 0.50 |
| Dairy | 900 -1200 | Drained for arable and grassland | 0.50 |
| LFA grazing | 900 -1200 | Free draining | 0.04 |
| LFA grazing | 900 -1200 | Drained for arable and grassland | 0.31 |
| LFA grazing | 1200 - 1500 | Free draining | 0.46 |
| LFA grazing | 1200 - 1500 | Drained for arable and grassland | 0.12 |
| LFA grazing | > 1500 | Free draining | 0.08 |
| Lowland grazing | 900 -1200 | Free draining | 0.90 |
| Lowland grazing | 900 -1200 | Drained for arable and grassland | 0.10 |
| Mixed | 900 -1200 | Free draining | 0.83 |
| Mixed | 1200 - 1500 | Free draining | 0.17 |

Table S5: Monitored average monthly (2000 – 2017) flow and concentrations of total suspended solids, total phosphorus and orthophosphate at the outlet monitoring station for the study catchment.

| Month | Total suspended solids | Total phosphorus | Orthophosphate | Flow rate |
| --- | --- | --- | --- | --- |
|  | (mg L^-1^) | (mg L^-1^) | (mg L^-1^) | m^3^ s^-1^ |
| Jan | 40.0 | 0.142 | 0.076 | 3.83 |
| Feb | 48.7 | 0.172 | 0.088 | 3.29 |
| Mar | 21.7 | 0.160 | 0.133 | 2.11 |
| Apr | 4.3 | 0.180 | 0.161 | 1.33 |
| May | 10.6 | 0.195 | 0.163 | 1.01 |
| Jun | 6.8 | 0.571 | 0.562 | 0.69 |
| Jul | 5.2 | 0.440 | 0.415 | 0.69 |
| Aug | 4.8 | 0.706 | 0.669 | 0.56 |
| Sep | 4.1 | 0.646 | 0.589 | 0.61 |
| Oct | 13.6 | 0.320 | 0.254 | 1.78 |
| Nov | 11.0 | 0.113 | 0.075 | 3.12 |
| Dec | 35.7 | 0.101 | 0.050 | 3.69 |

Table S6: Results of non-parametric pairwise comparisons of the predicted multiple impacts of the two intervention scenarios by soil type in the study area.

|  | p-value @ 0.05 confidence level |  |  |  |
| --- | --- | --- | --- | --- |
| Modelled outcome | Free draining soils |  | Drained soils |  |
| Reduction in nitrate loss | 0.0024 | TRUE | 0.7813 | FALSE |
| Reduction in phosphorus loss | 0.0015 | TRUE | 0.5781 | FALSE |
| Reduction in sediment loss | 0.0068 | TRUE | 0.2188 | FALSE |
| Reduction in N_2_O emissions | 0.0005 | TRUE | 0.0156 | TRUE |
| Reduction in FIO loss | 0.5845 | FALSE | 0.1563 | FALSE |
| Change in soil carbon stock | 1.0000 | FALSE | 0.7656 | FALSE |
| Chane in energy use | 0.7656 | FALSE | 1.0000 | FALSE |
| Reduction in pesticide loss | 0.0005 | TRUE | 1.0000 | FALSE |

Framing of modelled intervention scenarios

Farm structure and management data; environment data

Structure and

Modelled evaluation of scenario efficacies at farm scale relative to BAU

Modelled evaluation of scenario efficacies at landscape scale relative to BAU

Corrected scenario efficacies for emissions to water at landscape scale

Study catchment routine flow and water quality monitoring data

Estimated contributions from non-agricultural sources

Field monitoring and research on farm platform

Figure S1: Workflow summary.


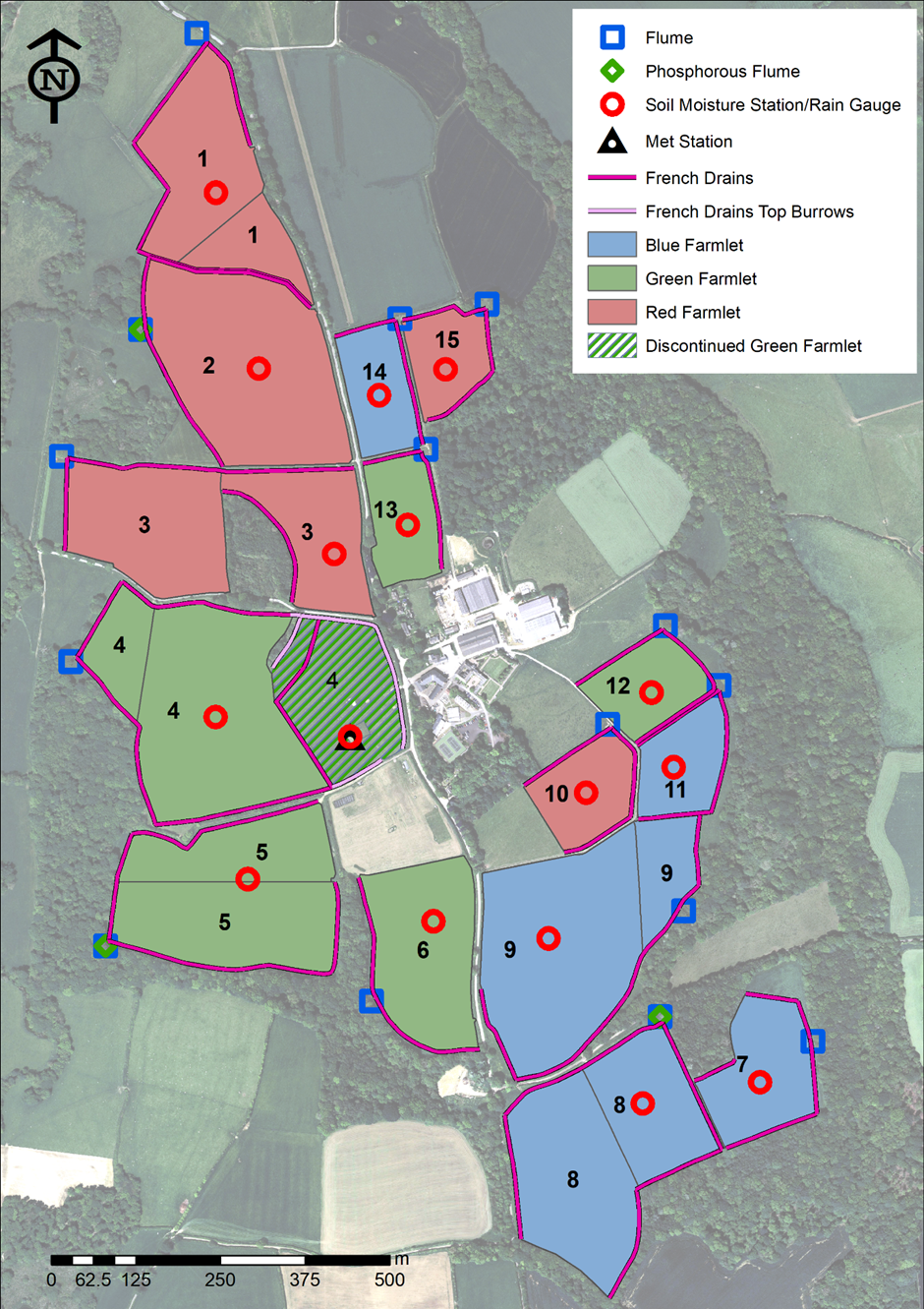


Figure S2: The North Wyke Farm Platform (NWFP) showing the hydrologically-isolated field scale catchments, three farmlets and locations of the key monitoring infrastructure.

**Module 1**

Environment data (rainfall, soil drainage status/type)

**Module 1**

Process-based model export coefficients for diffuse pollution from agricultural land to water (N, P, sediment, plant protection products, FIOs) and air (methane, nitrous oxide, ammonia)

**Module 1**

Robust farm type specific data on farm structures (cropping, livestock) and locations

**Module 1**

Integration of model outputs

**Module 2**

Scaling out within the agricultural sector for BAU landscape scale computation

**Module 1**

Existing best management practise uptake due to the combination of regulation, incentivisation and advice

**Module 1**

Business-as-usual (BAU) loadings of multiple pollutants to water (P, N, sediment, pesticides, FIOs) and air (methane, nitrous oxide, ammonia), plus additional outcomes for co-benefits or trade-offs – soil carbon, biodiversity, energy use, soil quality, farm economics

**Module 2**

Scaling out within the agricultural sector for intervention scenario landscape scale computation

**Module 3**

Corrected efficacy for impacts on total pollutant loadings to water for spatial mismatch

**Module 3**

Scaling up - cross-sector loads of multiple water pollutants

**Module 4**

Scaling up - categorisation of timeliness of landscape scale response to on-farm interventions for temporal mismatch

**Module 4**

Evaluation of slope to channel connectivity, floodplain buffering, hydromorphological modifications

**Module 5**

LCA mid-point impact categories – EP, AP, GWP20, GWP100, SP

Figure S3: Overview of the five modules in the Catchment Systems Model (CSM).

***
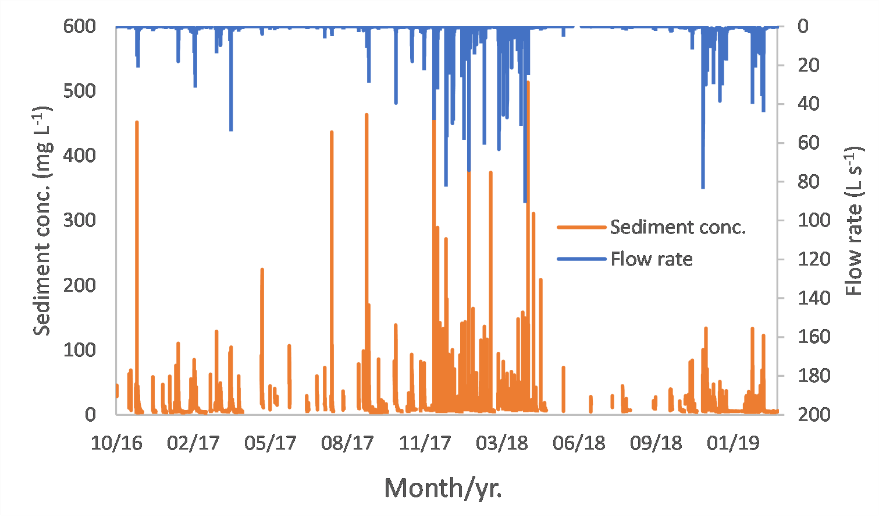
 (a)***

***
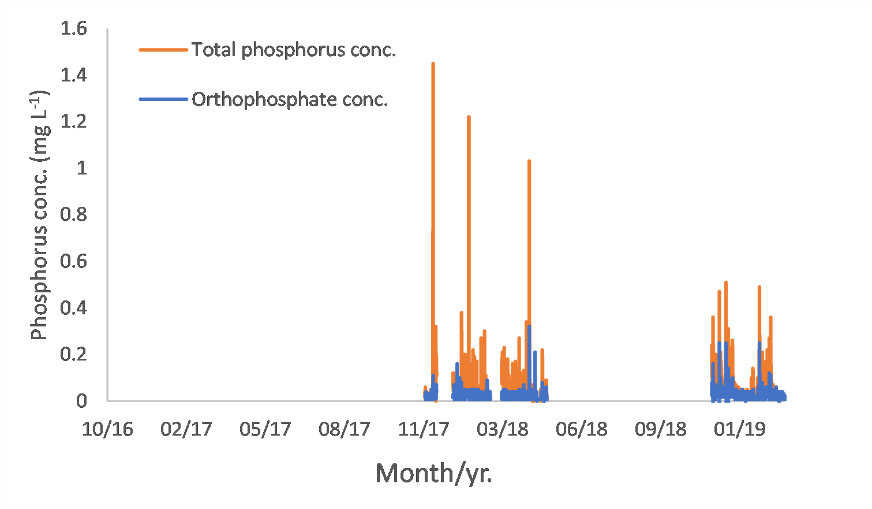
 (b)***


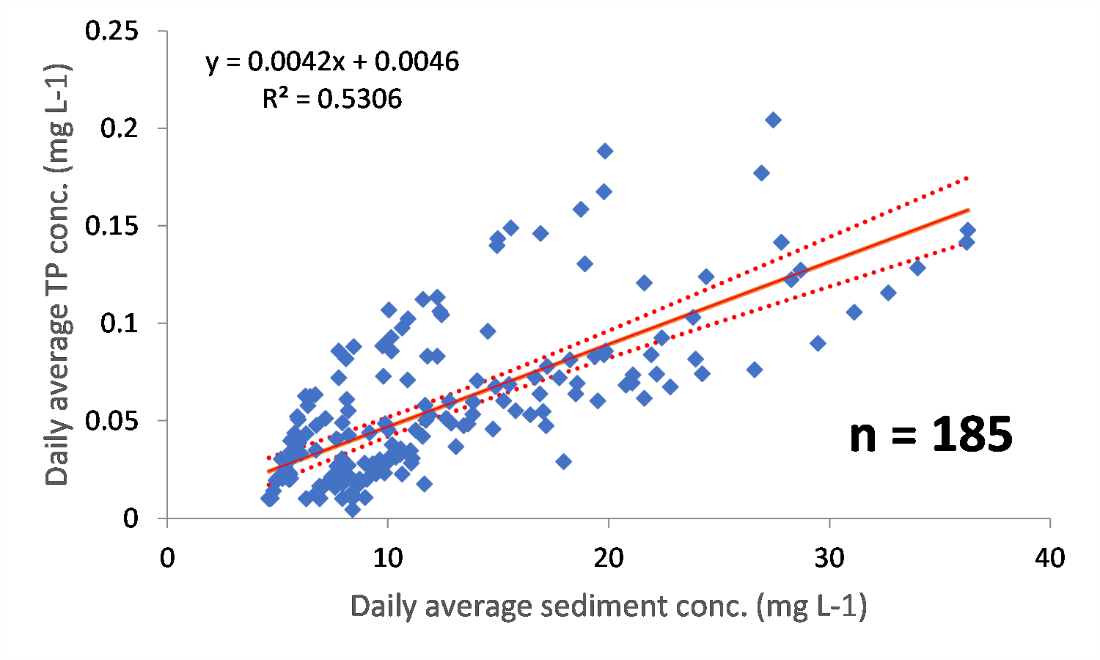
 ***(c)***

Figure S4: Monitored 15-minute flow and sediment concentrations (a); 15-minute orthophosphate (OP) and total phosphorus (TP) concentrations (b); the relationship between daily average concentrations of sediment and TP for NWFP field scale catchment 3 (c). In (c), the solid line is the fitted regression line and the dashed lines are the 95% confidence intervals.

***
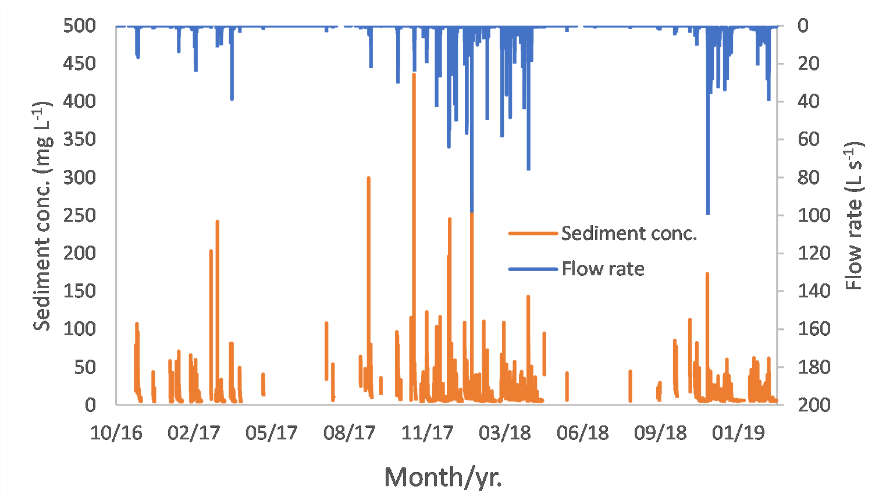
 (a)***

***
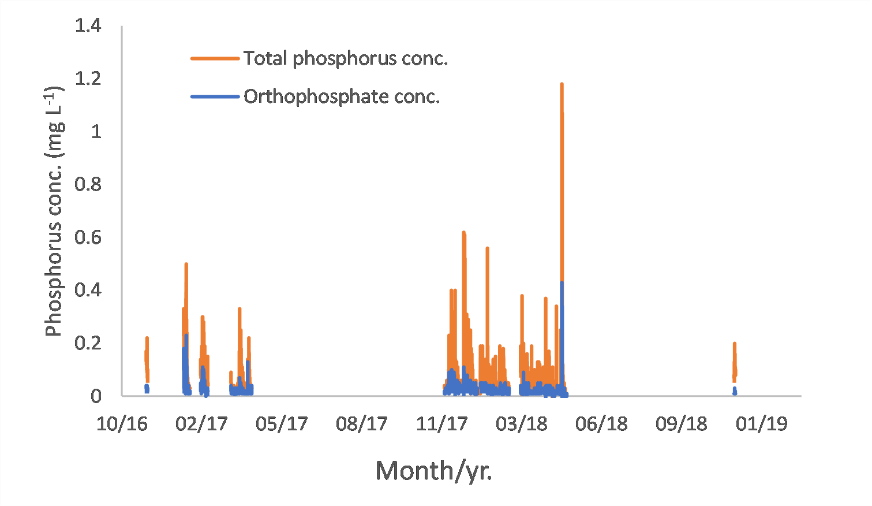
 (b)***


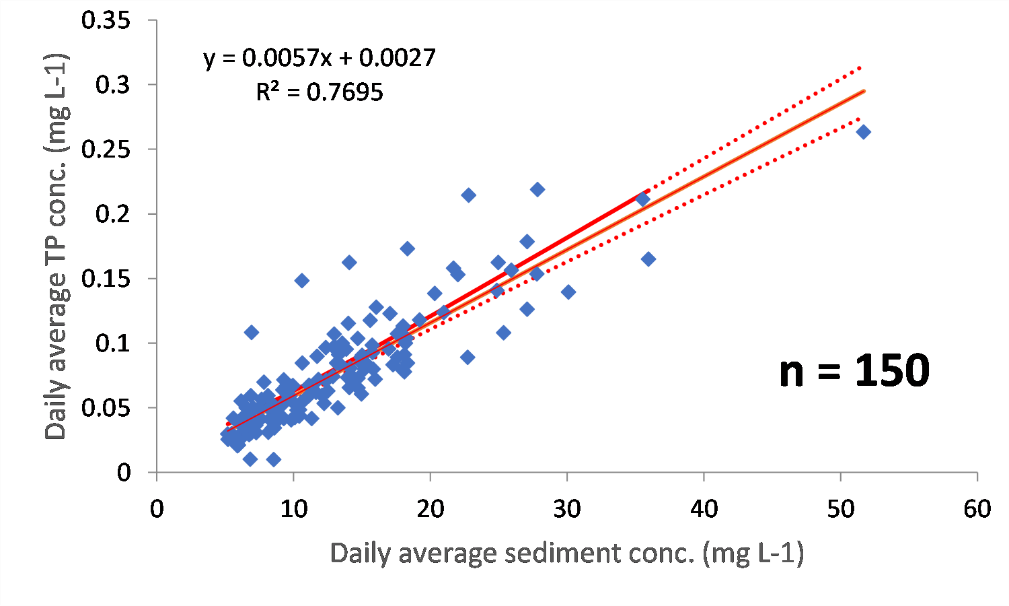
 ***(c)***

Figure S5: Monitored 15-minute flow and sediment concentrations (a); 15-minute orthophosphate (OP) and total phosphorus (TP) concentrations (b); relationship between daily average concentrations of sediment and TP for NWFP field scale catchment 2 (c). In (c), the solid line is the fitted regression line and the dashed lines are the 95% confidence intervals.

***
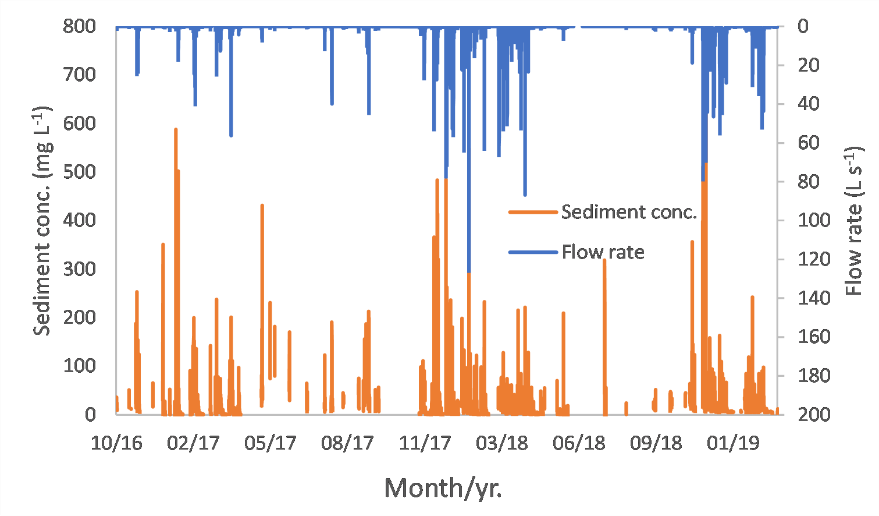
 (a)***

***
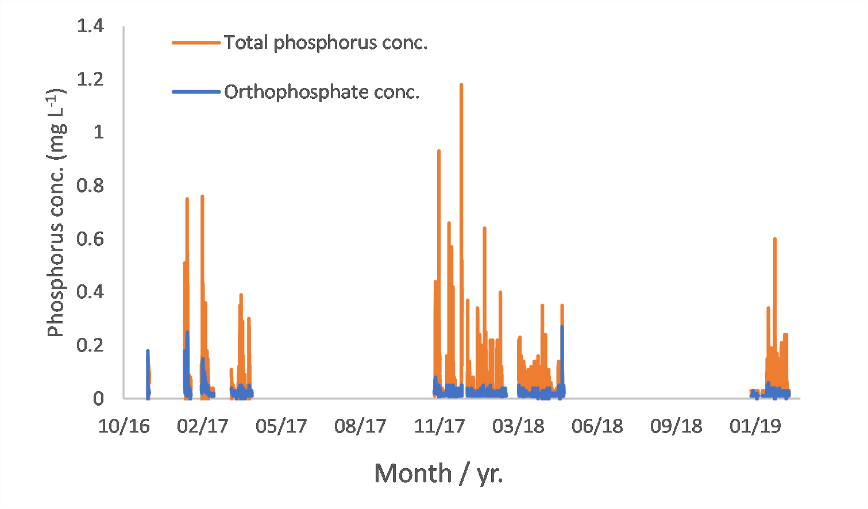
(b)***


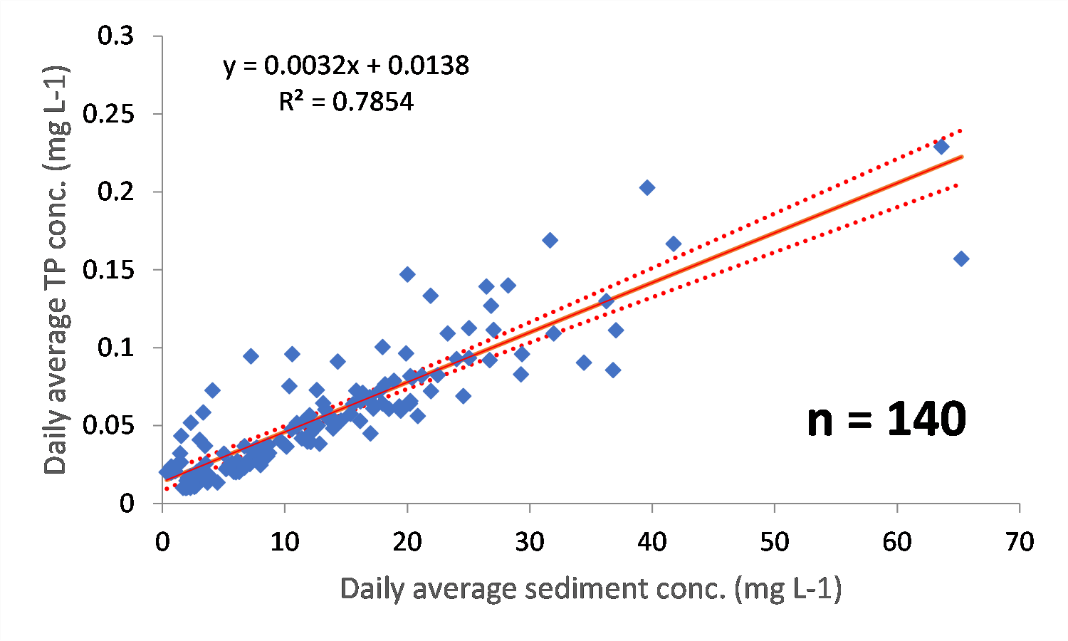
 ***(c)***

Figure S6: Monitored 15-minute flow and sediment concentrations (a); 15-minute orthophosphate (OP) and total phosphorus (TP) concentrations (b); relationship between daily average concentrations of sediment and TP for NWFP field scale catchment 8 (c). In (c), the solid line is the fitted regression line and the dashed lines are the 95% confidence intervals.

**Processing of monitored water quality data at field and landscape scales**

All data points with flagged known issues were removed from the time series. Further checks were made using visual comparison of the temporal trends between rainfall, flow and pollutant concentrations since at field scale, all of these parameters will be strongly correlated. Following the removal of erroneous values (e.g., sudden increases in flow or pollutant concentrations without recorded rainfall, singular extreme values of flow and pollutant concentrations including zero values, or static singular values of flow and pollutant concentrations during rainfall events), linear interpolation using two neighbouring valid data points was employed to fill any missing flow values. A calibration curve was established between paired monitored turbidity values (NTUs) and total suspended solids (TSS) measured on physically collected drainage water samples (filtration through 1.2 µm pore size, Whatman GFC filter papers and subsequent drying at 105°C). Corresponding estimates of suspended sediment concentrations were then calculated to estimate suspended sediment loss at the flumes on the NWFP (Peukert et al., 2014; Pulley and Collins, 2019). Quality assurance procedures revealed that valid results were obtained across the different NWFP field scale catchments for > 99% of the sampling times for discharge, 70 – 84% of sampling times for OP and TP, and 75- 92% of sampling times for suspended sediment.

**Water quality load estimation algorithms**

$L=K\left[\sum_{i=1}^{n} \frac{C_{i}+C_{i+1}}{2}\right]Q_{i}$ (1)

$L=K\left[\sum_{i=1}^{n} \frac{C_{i}+C_{i+1}}{2}\right]\mathop{Q_{p}}\limits^{-}$ (2)

$L=K\left[\sum_{i=1}^{n} \frac{C_{i}+C_{i+1}}{2}\right]\mathop{Q_{r}}\limits^{-}$ (3)

where:

$L$= load (kg or tonnes)

$K$= a conversion factor to account for a) time period and b) units

$C_{i}$= concentration at sampling time (mg L^-1^)

$Q_{i}$= discharge at sampling time (m^3^ s^-1^)

$\mathop{Q}\limits^{-} _{r}$= mean flow for period of load estimate (derived from a continuous discharge record (m^3^ s^-1^)

$\mathop{Q}\limits^{-} _{p}$= mean flow over the period between water samples (derived from a continuous discharge record (m^3^ s^-1^)

$n$= number of water quality samples collected / number of days on which concentrations were determined

**Further background on the farm scale module and its application in the study area**

Export coefficients for different farm types are based on process-based simulations derived from the PSYCHIC (Phosphorus and Sediment Yield CHaracterisation In Catchments) model (Collins et al., 2007, 2008. 2009; Collins and Anthony, 2008; Comber et al., 2013; Davison et al., 2008; Stromqvist et al., 2008) for sediment and P, NEAP-N (Lord and Anthony, 2000; Silgram et al., 2001) for nitrate, NARSES (National Ammonia Reduction Strategy Evaluation System; Webb and Misselbrook, 2004) and NT26AE (Chadwick et al., 2005) for ammonia emissions, IPCC (2006) for methane emissions and the IPCC methodology for nitrous oxide emissions from fertilisers, excreta and managed manure. Losses of faecal indicator organisms (FIOs) are based on the FIO-FARM model (Anthony and Morrow, 2011), whereas losses of pesticides in runoff or leaching are based on the MACRO (Jarvis, 1994) and SWAT (Brown and Hollis, 1996) tools, using publicly available data on application rates to different crop types (e.g. Garthwaite, 2006) and soil and climate relevant sorption capacities and degradation rates. Soil carbon predictions assume land is in equilibrium and are based on a soil stock (t ha^-1^; Bradley et al., 2005) approach to 1 m depth using an augmented IPCC Tier 1 methodology (Egglestone et al., 2006). Baseline estimates of energy (kg of CO_2_ equivalent) use are modified as a percentage change during on-farm mitigation scenarios taking account of energy use associated with major farm building and field operations (e.g., Antonio and Gloria 2010; Gibbons et al., 2004) and embedded emissions associated with fertilizer and pesticide production (IPCC 2006). Qualitative mitigation scenario impacts on terrestrial biodiversity, relative to BAU, are estimated using previous work on the impacts of agri-environment measures on key soil taxonomic groups (Boatman et al., 2008, 2010). Similarly, qualitative impacts on soil physical quality, representing connected porosity and water holding capacity are estimated using previous work on expert opinion of management measure relative impacts on compaction and associated soil functions (Balshaw et al., 2013; Rickson et al., 2010).

To account for the contributions of a small number of sheep on the upland in the headwaters, the uppermost portion of the study catchment was further divided into two areas: one with an AAR > 1500 mm, named as high rainfall moorland, and the other with an AAR between 1200 - 1500 mm, named as the low rainfall moorland (Figure 1). These two areas were represented in CSM using two virtual farms populated with estimated sheep numbers based on a mapped version of the 2010 JAS (Comber et al., 2008) and were classified as LFA grazing livestock farms. These were not subject to mitigation but only included for the estimation of the total catchment scale agricultural pollutant loadings delivered to the study landscape outlet.

**Further background on the grazing farms in the study area**The Upper Taw catchment is situated in Devon England, which is a major repository of farm animal diversity. It is home to two breeds of cattle (Devon and South Devon), five sheep breeds (Devon Closewool, Devon and Cornwall Longwool, Exmoor Horn, Greyface Dartmoor and White Face Dartmoor) and two pony breeds (Dartmoor and Exmoor).

To meet the demand for lower prices with less concern for quality, the beef industry in the area switched to larger cattle that can be reared quickly to produce lean meat in bulk mainly from commercial concentrate feeds since the 1960s (Wilson, 2011). These larger breeds were imported from continental Europe and rapidly displaced the old standard breeds. For example, the North Wyke Research Farm has 360 Suffolk x ewes and a 120-head beef suckler herd (currently both Stabiliser (ST) and Hereford (HE) X cattle but moving to a pure Stabiliser herd). Cattle population data for the whole waterbody within which the upper River Taw observatory resides for 2016 is shown below:

| **Cattle categories** | **Count** |
| --- | --- |
| Male Cattle under a year | 766 |
| Beef Female Cattle under a year | 496 |
| Dairy Female Cattle under a year | 157 |
| Male Cattle 1 - 2 years | 581 |
| Beef Female Cattle 1 - 2 years | 409 |
| Dairy Female Cattle 1 - 2 years | 108 |
| Male Cattle 2 years and over | 145 |
| Beef Females without offspring | 214 |
| Dairy Females without offspring | 113 |
| Beef Cows, Heifers that have calved and culled cows | 803 |
| Dairy Cows, Heifers that have calved and culled cows | 492 |

The sizes of the LFA grazing farms in the study area are typically ~82 ha. It is estimated that a higher proportion of cattle are on LFA grazing farms (46%) followed by dairy farms (27%) with the latter having a much higher stocking density (269 heads/km^2^ vs 92 heads /km^2^). As for sheep and lambs, these are predominantly on LFA grazing (61%) and lowland grazing farms (24%) with estimated respective stocking densities of 471 heads/km^2^ and 634 heads/ km^2^, respectively.

The Cattle Farm Practices Survey in 2019 suggests that the southwest region of England where the upper River Taw observatory is located is mainly (88%) a mix of housed grazing systems. Above the national average (38% vs 29%) of local livestock farms spread slurry on either arable or grazing land. About 36% of local livestock farms also store slurry for at least 6 months before application to land.

The table below summarises the best management interventions currently implemented on LFA grazing farms in the study area and their uptake rates under business-as-usual (BAU):

| **Intervention** | **Free draining** | **Drained soil** |
| --- | --- | --- |
| Additional targeted bedding for straw-bedded cattle housing | 10 | 10 |
| Adopt phase feeding of livestock | 80 | 80 |
| Adopt reduced cultivation systems | 2 | 25 |
| Allow cattle slurry stores to develop a natural crust | 80 | 80 |
| Anaerobic digestion of livestock manures | 2 | 2 |
| Avoid irrigating at high risk times | 25 | 2 |
| Avoid PPP application at high risk timings | 25 | 10 |
| Avoid spreading manufactured fertiliser to fields at high-risk times | 2 | 2 |
| Calibration of sprayer | 50 | 50 |
| Capture of dirty water in a dirty water store | 50 | 50 |
| Compost solid manure | 20 | 20 |
| Construct bridges for livestock crossing rivers/streams | 25 | 25 |
| Construct bunded impermeable PPP filling/mixing/cleaning area | 2 | 2 |
| **Construct troughs with a concrete base** | **2** | **2** |
| Cover solid manure stores with sheeting | 2 | 2 |
| Cultivate and drill across the slope | 25 | 10 |
| Cultivate compacted tillage soils | 10 | 10 |
| Cultivate land for crops in spring rather than autumn | 80 | 2 |
| Ditch management on arable land | 0 | 50 |
| Ditch management on grassland | 0 | 25 |
| Do not apply manufactured fertiliser to high-risk areas | 50 | 50 |
| Do not apply manure to high-risk areas | 80 | 80 |
| Do not apply P fertilisers to high P index soils | 50 | 50 |
| Do not spread FYM to fields at high-risk times | 2 | 2 |
| Do not spread slurry or poultry manure at high-risk times | 2 | 2 |
| Drift reduction methods | 25 | 25 |
| Early harvesting and establishment of crops in the autumn | 50 | 50 |
| Establish and maintain artificial wetlands - steading runoff | 0 | 2 |
| Establish cover crops in the autumn | 2 | 0 |
| Establish in-field grass buffer strips | 2 | 2 |
| Establish new hedges | 2 | 2 |
| **Establish riparian buffer strips** | **10** | **10** |
| Establish tree shelter belts around livestock housing | 10 | 10 |
| Extend the grazing season for cattle | 10 | 10 |
| **Manage farm tracks** | **25** | **25** |
| Fence off rivers and streams from livestock | 25 | 25 |
| Fertiliser spreader calibration | 50 | 50 |
| Fill/Mix/Clean sprayer in field | 25 | 25 |
| Frequent removal of slurry from beneath-slat storage in pig housing | 2 | 2 |
| Incorporate manure into the soil | 2 | 2 |
| Increase scraping frequency in dairy cow cubicle housing | 10 | 10 |
| Increase the capacity of farm slurry stores to improve timing of slurry applications | 10 | 10 |
| In-house poultry manure drying | 10 | 10 |
| Install air-scrubbers or biotrickling filters in mechanically ventilated pig housing | 2 | 2 |
| Install covers to slurry stores | 10 | 10 |
| Integrate fertiliser and manure nutrient supply | 50 | 50 |
| Irrigate crops to achieve maximum yield | 25 | 2 |
| Irrigation/water supply equipment is maintained and leaks repaired | 50 | 10 |
| Leave autumn seedbeds rough | 10 | 10 |
| Leave over winter stubbles | 80 | 2 |
| Leave residual levels of non-aggressive weeds in crops | 2 | 2 |
| **Locate out-wintered stock away from watercourses** | **50** | **50** |
| **Loosen compacted soil layers in grass fields** | **10** | **10** |
| Make use of improved genetic resources in livestock | 10 | 10 |
| Manage over-winter tramlines | 10 | 10 |
| Management of field corners | 10 | 10 |
| Manure Spreader Calibration | 25 | 25 |
| Minimise the volume of dirty water produced (sent to dirty water store) | 10 | 10 |
| Minimise the volume of dirty water produced (sent to slurry store) | 10 | 10 |
| Monitor and amend soil pH status for grassland | 50 | 50 |
| More frequent manure removal from laying hen housing with manure belt systems | 10 | 10 |
| **Move feeder rings at regular intervals** | **50** | **50** |
| Plant areas of farm with wild bird seed / nectar flower mixtures | 10 | 10 |
| Protection of in-field trees | 10 | 10 |
| Reduce dietary N and P intakes: Dairy | 10 | 10 |
| Reduce dietary N and P intakes: Pigs | 80 | 80 |
| Reduce dietary N and P intakes: Poultry | 80 | 80 |
| **Reduce field stocking rates when soils are wet** | **65** | **65** |
| **Reduce the length of the grazing season** | **10** | **10** |
| Replace urea fertiliser to arable land with another form | 25 | 25 |
| **Re-site gateways away from high risk areas** | **0** | **0** |
| Site solid manure heaps away from watercourses/field drains | 95 | 80 |
| Store solid manure heaps on an impermeable base and collect effluent | 10 | 10 |
| Treatment of PPP washings through disposal, activated carbon or biobeds | 50 | 50 |
| Use a fertiliser recommendation system | 80 | 80 |
| Use clover in place of fertiliser nitrogen | 30 | 30 |
| **Use correctly inflated low ground pressure tyres** | **25** | **25** |
| Use efficient irrigation techniques (boom trickle, self closing nozzles) | 10 | 0 |
| Use high sugar grasses | 15 | 15 |
| Use liquid/solid manure separation techniques | 2 | 2 |
| Use manufactured fertiliser placement technologies | 10 | 10 |
| Use plants with improved nitrogen use efficiency | 10 | 10 |
| Use slurry band spreading application techniques | 2 | 2 |
| Washing down of dairy cow collecting yards | 25 | 25 |

**References**

Anthony S.G. and Morrow, K., 2011. Prototype farm scale faecal indicator budget model. Final Report for Defra Project WQ0111, 89 pp.

Antonio M. A. and Gloria J. G., 2010. Comparison of the Efficiency and Use of Energy in Organic and Conventional Farming in Spanish Agricultural Systems. Journal of Sustainable Agriculture 34(3), 312-338, DOI: 10.1080/10440041003613362

Balshaw, H., Newell-Price, P., Ctitchley, N., Harris, D., Twining, S, Chambers, B., 2013. Post harvest management for soil degradation reduction in agricultural soils: methods, occurrence, cost and benefits. Final Report for Defra Project SP1315.

Boatman, N., Conyers, S., Parry, H., Pietravalle, S., Ramwell, C., 2008. Estimating impacts of ELS on key biodiversity indicators and diffuse pollution of surface waters by nutrients. Final Report for Defra Project MA01041.

Boatman, N., Willis, K., Garrod, G., Powe, N., 2010. Estimating the wildlife and landscape benefits of Environmental Stewardship. Final Report for Natural England Project RP0025.

Brown, C. and Hollis, J. 1996. SWAT – a semi-empirical model to predict concentrations of pesticides entering surface waters from agricultural land. Pesticide Science, 47, 41-50.

Chadwick, D., Misselbrook, T., Gilhespy, S., Williams, J., Bhogal, A., Sagoo, L., Nicholson, F., Webb, J., Anthony, S., Chambers, B., 2005. Ammonia emissions and crop nitrogen use efficiency: ammonia emissions from nitrogen fertiliser applications to grassland and tillage land; Factors affecting ammonia emissions from urea based fertilisers; and Ammonia emissions model., Final Report for Defra Project NT2605, p. 71.

Collins, A.L. and Anthony, S.G., 2008. Assessing the likelihood of catchments across England

and Wales meeting ‘good ecological status’ due to sediment contributions from agricultural

sources. Soil Use and Management 11, 163–170.

Collins, A.L., Stromqvist, J., Davison, P.S., Lord, E.I., 2007. Appraisal of phosphorus and sediment transfer in three pilot areas identified for the Catchment Sensitive Farming initiative

in England: application of the prototype PSYCHIC model. Soil Use and Management 23,

117–132.

Collins, A.L., Duethmann, D. and Taylor, P., 2008. National-scale sediment delivery to watercourses across England and Wales under recent (1970-2004) land use change. In: *Sediment dynamics in changing environments* (pp 448-452), International Association of Hydrological Sciences Publication No. 325, Wallingford, UK.

Collins, A.L., Anthony, S.G., Hawley, J., Turner, T., 2009. The potential impact of projected

change in farming by 2015 on the importance of the agricultural sector as a sediment

source in England and Wales. Catena 79, 243–250.

Comber, A., Anthony, A., Proctor, C., 2008. The creation of a national agricultural land use dataset: combining pycnophylactic interpolation with dasymetric mapping techniques. Transactions in GIS 12(6), 775–791.

Davison, P.S., Withers, P.J.A., Lord, E.I., Betson, M.J., Stromqvist, J., 2008. PSYCHIC — a

process-based model of phosphorus and sediment mobilization and delivery within agricultural catchments in England and Wales: Part 1 -model description and parameterization.

Journal of Hydrology 350, 290–302.

Department for Environment, Food and Rural Affairs (Defra), 2019. Cattle Farm Practices Survey 2019 England. Available at <https://www.gov.uk/government/statistics/cattle-farm-practices-survey-april-2019> and accessed on 9th Aug 2020.

Eggleston, S., Buendia, L., Miwa, K., Ngara, T. and Tanabe K., 2006. Guidelines of National Greenhouse Gas Inventories. Volume 4. Agriculture, Forestry and Other Land Use. IPCC.

Garthwaite, D., Thomas, M., Heywood, E. and Battersby, A., 2006. Pesticide usage surface report 213 – Arable crops in Great Britain 2006. Pesticide Usage Survey Team, Central Science Laboratory, York, 120 pp.

Gibbons, M.M., Anthony, S.G., and Smith, K.A., 2004. SPREADS – A system for controlling the costs and efficiency of manure and slurry spreading on farms. In: Bernal, M.A., Moral, R., Clemente, R. Paredes, C. (Eds.) Proceedings of the 11th International Conference of the FAO ESCORENA on the recycling of agriculture, municipal and industrial residues in agriculture (RAMIRAN 2004), Murcia, Spain, pp. 349–352.

IPCC, 2006. In: Eggleston, H.S., Buendia, L., Miwa, K., Ngara, T., Tanabe, K. (Eds.), IPCC Guidelines for National Greenhouse Gas Inventories. Agriculture, Forestry and Other Land Use Vol. 4. Institute for Global Environmental Strategies, Japan, pp. 256–267.

Jarvis, N., 1994. The MACRO model Version 3.1 — Technical description and sample simulation. In: Reports and Dissertations 19, Dept. Soil Sci., Swedish Univ. Agric. Sci. (1994), p. 51.

Lord, E. and Anthony, S., 2000. MAGPIE: a modelling framework for evaluating nitrate losses

at national and catchment scales. Soil Use and Management 16, 167–174.

Rickson, J., Deeks, L., Posthumous H. and Quinton, J. 2010. Sub-Project C of Defra Project SP1601: Soil Functions, Quality and Degradation – Studies in Support of the Implementation of Soil Policy. Defra, London, UK.

Silgram, M., Waring, R., Anthony, S., Webb, J., 2001. Intercomparison of national and IPCC

methods for estimating N loss from agricultural land. Nutrient Cycling in Agroecosystems 60, 189–195.

Stromqvist, J., Collins, A.L., Davison, P.S., Lord, E.I., 2008. PSYCHIC— a process-based model of phosphorus and sediment transfers within agricultural catchments. Part 2. A preliminary evaluation. Journal of Hydrology 350, 303–316.

Webb, J. and Misselbrook, T., 2004. A mass flow model of ammonia emissions from UK livestock production. Atmospheric Environment 38 (14), 2163–2176.

Wilson, R.T., 2011. The indigenous domestic livestock of the County of Devon, England. Animal Genetic Resources, 47, 63–77. © Food and Agriculture Organization of the United Nations, 2011 doi:10.1017/S2078633610001037.
